# Supplementary material for: Dissipative realization of Kondo models
Source: Commun Phys. 2025 May 22;8(1):212. doi: 10.1038/s42005-025-02141-x (PMC12098120; doi:10.1038/s42005-025-02141-x)
Supplement: Supplementary file 2 — Supplementary Material [file 42005_2025_2141_MOESM2_ESM.pdf]

Supplementary Material — Dissipative realization of Kondo models

Martino Stefanini[1], Yi-Fan Qu[2], Tilman Esslinger[3], Sarang Gopalakrishnan[4], Eugene Demler[2], Jamir Marino[1]

[1]Institut für Physik, Johannes Gutenberg-Universität Mainz, Mainz, D-55099, Germany.
[2]Institute for Theoretical Physics, ETH Zürich, Zurich, 8093, Switzerland.
[3]Institute for Quantum Electronics & Quantum Center, ETH Zürich, Zurich, 8093, Switzerland.
[4]Department of Electrical and Computer Engineering, Princeton University, Princeton, 08544, New Jersey, USA.

Supplementary Note 1: Derivation of the effective dynamics

In this Section we derive the effective master equation governing the dynamics of the single-site dissipative dot at large dissipation.

We consider the strongly dissipative limit in which the loss rate is the largest energy scale $\gamma \gg W, |\varepsilon_d|, \Gamma$. In other words, we will take the purely dissipative dynamics as the zeroth-order solution and expand in the Hamiltonian contribution to the Lindblad equation. This approach is slightly different from the usual Schrieffer-Wolff mapping of the Anderson impurity model to the Kondo model [1, 2], which treats the uncoupled dot-leads system as the unperturbed dynamics and expands in H_{tun}, i.e. in Γ. In the present model, this would amount to $\gamma \sim W \sim |\varepsilon_d| \gg \Gamma$. Although it is possible to adopt this approach also in the dissipative case, it yields considerably more complicated expressions for the effective dynamics, while adding very little to the physical description of the system.

The starting point of our analysis is the purely dissipative dot site, decoupled from the rest of the system. The master equation becomes

$$\frac{\mathrm{d}}{\mathrm{d}t}\rho = \mathcal{L}_0 \rho \ , \tag{1}$$

where

$$\mathcal{L}_0 \rho \equiv \gamma\big(L\rho L^\dagger - \tfrac{1}{2}\{L^\dagger L, \rho\}\big) \tag{2}$$

We label the states of the dissipative site as $|\alpha\rangle$, where $\alpha \in \{0, \uparrow, \downarrow, d = \uparrow\downarrow\}$, and vectorize the density matrix as [3] $\rho = \sum_{\alpha\beta} \rho_{\alpha\beta}\, |\alpha\rangle\langle\beta| \to |\rho) = \sum_{\alpha\beta} \rho_{\alpha\beta} |\alpha\beta)$. Then, it is easy to see that in the 16-dimensional $|\alpha\beta)$ basis the Liouville superoperator $\mathcal{L}_0$ is almost diagonal:

$$\begin{aligned}
&\mathcal{L}_0|\alpha\beta) = 0 \quad \text{for all } \alpha,\ \beta \in \{0,\uparrow,\downarrow\} \ ,\\
&\mathcal{L}_0|\alpha d) = -\frac{\gamma}{2}|\alpha d) \quad \text{for all } \alpha \in \{0,\uparrow,\downarrow\} \ ,\\
&\mathcal{L}_0|d\alpha) = -\frac{\gamma}{2}|d\alpha) \quad \text{for all } \alpha \in \{0,\uparrow,\downarrow\} \ ,\\
&\mathcal{L}_0|dd) = \gamma(|00) - |dd))
\end{aligned}$$

The only coupled states are $|00)$ and $|dd)$, and the diagonalization of the corresponding 2×2 matrix yields the remaining two eigenvalues of $\mathcal{L}_0$:

$$\left.\begin{aligned}
&\mathcal{L}_0|\phi_0^R) = 0, \quad |\phi_0^R) = |00)\\
&(\phi_0^L|\mathcal{L}_0 = 0, \quad (\phi_0^L| = (00| + (dd|
\end{aligned}\right\} \quad \lambda_0 = 0 \tag{3a}$$

$$\left.\begin{aligned}
&\mathcal{L}_0|\phi_d^R) = -\gamma|\phi_d^R), \quad |\phi_d^R) = -|00) + |dd)\\
&(\phi_d^L|\mathcal{L}_0 = -\gamma(\phi_d^L|, \quad (\phi_d^L| = (dd|
\end{aligned}\right\} \quad \lambda_2 = -\gamma \tag{3b}$$

As usual, we are normalizing the states as $(\phi_a^L|\phi_b^R) = \delta_{ab}$. Summing up, there are nine stationary (dark) states with eigenvalue $\lambda_0 = 0$, six states with eigenvalue $\lambda_1 = -\frac{\gamma}{2}$, and a non-degenerate state with $\lambda_2 = -\gamma$. For later use, we report here the superprojectors on the three eigenspaces:

$$\mathcal{P}_0 \equiv |00)(dd| + \sum_{\alpha,\beta<d} |\alpha\beta)(\alpha\beta| \ , \tag{4a}$$

$$\mathcal{P}_1 \equiv \sum_{\alpha<d} (|\alpha d)(\alpha d| + |d\alpha)(d\alpha|) \ , \tag{4b}$$

$$\mathcal{P}_2 \equiv |dd)(dd| - |00)(dd| \tag{4c}$$

The $\mathcal{P}_0$ projector defines the slow subspace in which the dynamics will be confined at large dissipation. It is useful to express the above superprojectors in terms of operators in the ordinary Hilbert space:

$$\mathcal{P}_0 \rho = X_{0d}\, \rho\, X_{d0} + X_< \,\rho\, X_< \ , \tag{5a}$$

$$\mathcal{P}_1\rho = X_< \,\rho\, X_d + X_d \,\rho\, X_< \ , \tag{5b}$$
$$\mathcal{P}_2\rho = X_d \,\rho\, X_d - X_{0d} \,\rho\, X_{d0} \ , \tag{5c}$$

where we have introduced the ordinary projectors (Hubbard operators [1, 2, 4])

$$X_{\alpha\beta} \equiv |\alpha\rangle\langle\beta| \ , \tag{6a}$$
$$X_\alpha \equiv X_{\alpha\alpha} = |\alpha\rangle\langle\alpha| \ , \tag{6b}$$
$$X_< \equiv \sum_{\alpha<d} X_\alpha \tag{6c}$$

We turn to consider the full Lindblad master equation

$$\frac{\mathrm{d}}{\mathrm{d}t}\rho = (\mathcal{L}_0 + \mathcal{L}_H)\rho \ , \tag{7}$$

where $\mathcal{L}_H = -\mathrm{i}[H, \cdot]$ is the Hamiltonian part, which we will treat as a perturbation. Following [5] and [3], the effective dynamics in the slow subspace $\mathcal{P}_0\rho = \rho$ in the $\gamma \to +\infty$ limit is generated by the effective Lindblad superoperator $\mathcal{L}_{\text{eff}} = \mathcal{L}_1 + \mathcal{L}_2 + \mathcal{O}\big(\gamma^{-2}\big)$, where

$$\mathcal{L}_1 = \mathcal{P}_0 \mathcal{L}_H \mathcal{P}_0 \ , \tag{8a}$$
$$\mathcal{L}_2 = -\mathcal{P}_0 \mathcal{L}_H (\mathcal{Q}_0 \mathcal{L}_0 \mathcal{Q}_0)^{-1} \mathcal{L}_H \mathcal{P}_0 \ , \tag{8b}$$

and $\mathcal{Q}_0 = \mathbb{1} - \mathcal{P}_0$ is the complementary projector to $\mathcal{P}_0$. The first-order term is just the Hamiltonian part projected on the stationary subspace of $\mathcal{L}_0$, $\mathcal{L}_1\rho = \mathcal{P}_0\mathcal{L}_H\mathcal{P}_0\rho$. We classify the Hamiltonian terms according to the commutativity of the corresponding superoperator $-\mathrm{i}[H, \cdot]$ with $\mathcal{P}_0$. The dot Hamiltonian H_{dot} and the leads' one H_{leads} both commute (the latter since $\mathcal{P}_0$ acts as the identity on the leads states). The tunneling term H_{tun} is the only one which does not commute, since it connects the slow subspace $\mathcal{P}_0\rho = \rho$ with the other subspaces. The decomposition $d_\sigma = X_{0\sigma} + \sigma X_{\bar{\sigma}d}$ induces the further subdivision $H_{\text{tun}} = H^0_{\text{tun}} + H^1_{\text{tun}}$, with

$$\begin{aligned} H^0_{\text{tun}} &= \sum_\sigma (X_{\sigma 0}\Psi_\sigma + \Psi^\dagger_\sigma X_{0\sigma}) \ , \\ H^1_{\text{tun}} &= \sum_\sigma \sigma (X_{d\bar{\sigma}}\Psi_\sigma + \Psi^\dagger_\sigma X_{\bar{\sigma}d}) \ , \end{aligned} \tag{9}$$

where $\Psi_\sigma \equiv \sum_{p\alpha} V_{p\alpha} c_{p\sigma\alpha}$. Notice that the projectors $X_{\sigma 0}$, $X_{d\sigma}$ and their conjugates are of fermionic nature in the sense that they anticommute with Ψ_σ, $\Psi^\dagger_\sigma$, while all other projectors are bosonic (i.e. commuting with the Ψs). The first term H^0_{tun} commutes with $\mathcal{P}_0$, while the second is annihilated by it. So, if we call $H_0 = H_{\text{dot}} + H_{\text{leads}} + H^0_{\text{tun}}$ the part of the Hamiltonian that commutes with $\mathcal{P}_0$, we have

$$\mathcal{L}_1\rho = \mathcal{P}_0(\mathcal{L}_{H_0} + \mathcal{L}_{H^1_{\text{tun}}})\mathcal{P}_0\rho = \mathcal{L}_{H_0}\mathcal{P}_0^2\rho + \mathcal{P}_0\mathcal{L}_{H^1_{\text{tun}}}\mathcal{P}_0\rho = \mathcal{L}_{H_0}\rho \tag{10}$$

where we used $\rho = \mathcal{P}_0\rho$. The Hamiltonian H_0 is the Anderson impurity model with infinite dissipation quoted in Eq. (1) in the main text.

The second-order term $\mathcal{L}_2$ yields the effective dissipation. Since $\mathcal{Q}_0\mathcal{L}_0\mathcal{Q}_0 = -\gamma/2\mathcal{P}_1 - \gamma\mathcal{P}_2$, we have $(\mathcal{Q}_0\mathcal{L}_0\mathcal{Q}_0)^{-1} = -2/\gamma\,\mathcal{P}_1 - 1/\gamma\,\mathcal{P}_2$. We evaluate Eq. (8b) starting from its rightmost terms:

$$(\mathcal{Q}_0\mathcal{L}_0\mathcal{Q}_0)^{-1}\mathcal{L}_H\mathcal{P}_0\rho = \Big(-\frac{2}{\gamma}\mathcal{P}_1 - \frac{1}{\gamma}\mathcal{P}_2\Big)(\mathcal{L}_{H_0} + \mathcal{L}_{H^1_{\rm tun}})\rho = \frac{2}{\gamma}\mathcal{P}_1\mathcal{L}_{H^1_{\rm tun}}\rho\ . \tag{11}$$

In the expression above, the terms proportional to $\mathcal{L}_{H_0}\rho$ vanish because of the commutativity of $\mathcal{L}_{H_0}$ with both projectors $\mathcal{P}_{1,2}$, which entails $\mathcal{P}_{1,2}\mathcal{L}_{H_0}\mathcal{P}_0 = \mathcal{P}_{1,2}\mathcal{P}_0\mathcal{L}_{H_0} = 0$. The term proportional to $\mathcal{P}_2\mathcal{L}_{H^1_{\rm tun}}\rho$ also vanishes because if ρ belongs to the slow subspace $\mathcal{P}_0$, $\mathcal{L}_{H^1_{\rm tun}}\rho$ yields terms belonging to the $\mathcal{P}_1$ subspace, but not to the $\mathcal{P}_2$ one. Then we expand

$$\begin{aligned}
\frac{2}{\gamma}\mathcal{P}_1\mathcal{L}_{H^1_{\rm tun}}\rho &= \frac{2\mathrm{i}}{\gamma}\big(X_<H^1_{\rm tun}\rho X_d - X_<\rho H^1_{\rm tun}X_d + X_dH^1_{\rm tun}\rho X_< - X_d\rho H^1_{\rm tun}X_<\big)\\
&= \frac{2\mathrm{i}}{\gamma}\sum_\sigma \sigma\big[\Psi^\dagger_\sigma X_{\bar\sigma d}\rho X_d - X_<\rho\Psi^\dagger_\sigma X_{\bar\sigma d} + X_{d\bar\sigma}\Psi_\sigma\rho X_< - X_d\rho X_{d\bar\sigma}\Psi_\sigma\big]\\
&= \frac{2\mathrm{i}}{\gamma}\sum_\sigma \sigma\big[-X_<\rho\Psi^\dagger_\sigma X_{\bar\sigma d} + X_{d\bar\sigma}\Psi_\sigma\rho X_<\big]\ ,
\end{aligned}$$

where in the last equality we used $X_{\alpha d}\rho X_{d\beta} = 0$ for ρ in the slow subspace. Then,

$$\begin{aligned}
\mathcal{L}_2 &= -\mathcal{P}_0\big(\mathcal{L}_{H_0} + \mathcal{L}_{H^1_{\rm tun}}\big)\mathcal{P}_1\mathcal{L}_{H^1_{\rm tun}}\rho\\
&= -\frac{2}{\gamma}\mathcal{L}_{H_0}\mathcal{P}_0\mathcal{P}_1\mathcal{L}_{H^1_{\rm tun}} - \frac{2}{\gamma}\mathcal{P}_0\mathcal{L}_{H^1_{\rm tun}}\mathcal{P}_1\mathcal{L}_{H^1_{\rm tun}}\rho = -\frac{2}{\gamma}\mathcal{P}_0\mathcal{L}_{H^1_{\rm tun}}\mathcal{P}_1\mathcal{L}_{H^1_{\rm tun}}\rho\\
&= -\frac{2}{\gamma}\mathcal{P}_0\sum_{\sigma\tau}\sigma\tau\big(-2X_{d\bar\tau}\Psi_\tau\rho\Psi^\dagger_\sigma X_{\bar\sigma d} + X_<\rho\Psi^\dagger_\sigma X_{\bar\sigma\bar\tau}\Psi_\tau + \Psi^\dagger_\sigma X_{\bar\sigma\bar\tau}\Psi_\tau\rho X_<\big)\\
&= \frac{4}{\gamma}\sum_{\sigma\tau}\sigma\tau\big(X_{d\bar\tau}\Psi_\tau\rho\Psi^\dagger_\sigma X_{\bar\sigma d} - \frac{1}{2}\rho\Psi^\dagger_\sigma X_{\bar\sigma\bar\tau}\Psi_\tau - \frac{1}{2}\Psi^\dagger_\sigma X_{\bar\sigma\bar\tau}\Psi_\tau\rho\big)\ ,
\end{aligned}$$

where we recognize the dissipative part of the effective Lindblad superoperator,

$$\mathcal{L}_{\rm eff}\rho = L_{\rm eff}\rho L^\dagger_{\rm eff} - \tfrac{1}{2}\{L^\dagger_{\rm eff}L_{\rm eff}, \rho\} \tag{12}$$

with

$$L_{\rm eff} \equiv \frac{2}{\gamma^{1/2}}\sum_\sigma \sigma X_{0\bar\sigma}\Psi_\sigma = \frac{2}{\gamma^{1/2}}\sum_{p\alpha\sigma}\sigma V_{p\alpha}X_{0\bar\sigma}c_{p\sigma\alpha}\ , \tag{13}$$

which is the one quoted in the main text.

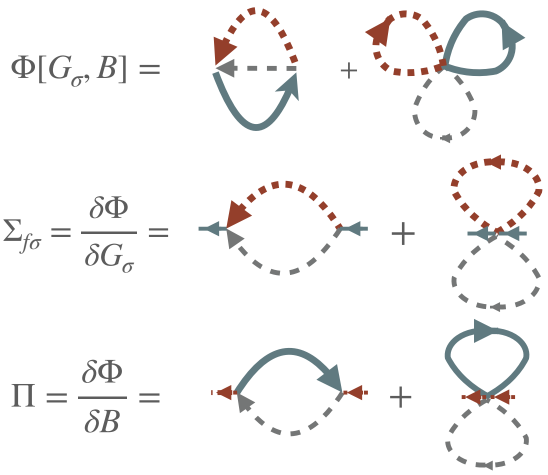

Supplementary Figure 1: Upper row: diagrammatic representation of the Luttinger-Ward functional truncated to the second order in the dot-leads hopping. The thick, aquamarine lines are the Green's functions of the auxiliary fermions, the dotted red lines are those of the auxiliary bosons and the dashed gray lines are the local ones of the leads. The lower rows of the figures depict the noninteracting fermionic and bosonic self-energies derived from Φ. In each self-energy, the first diagram is the usual NCA self-energy coming from the effective AIM Hamiltonian, while the second term is the mean-field contribution from the effective dissipation. The figures represent the diagrams before the projection onto the physical $Q = 1$ subspace.

Supplementary Note 2: Details on the non-crossing approximation

In this Section, we provide analytical details of our implementation of the non-crossing approximation and on our real-time calculations.

The Anderson impurity model with infinite repulsion that represents the effective unitary dynamics of our dissipative model is not suitable to the usual perturbative techniques based on Green's functions, since the Hubbard operators $X_{\alpha\beta}$ do not obey a standard fermionic or bosonic algebra and so their correlation functions cannot be extracted through Wick's theorem. So, we convert the effective Hamiltonian to a form that is suitable to standard perturbative treatments. This is achieved through the slave boson mapping of the Hubbard operators to a boson b and a spinful auxiliary fermion f_σ [1, 6, 7]:

$$
\begin{aligned}
X_{\sigma 0} &= |\sigma\rangle\langle 0| = d_\sigma^\dagger(\mathbb{1} - n_{\bar\sigma}) = f_\sigma^\dagger b \\
X_{0\sigma} &= |0\rangle\langle\sigma| = (\mathbb{1} - n_{\bar\sigma})d_\sigma = b^\dagger f_\sigma \\
X_{00} &= |0\rangle\langle 0| = (\mathbb{1} - n_\uparrow)(\mathbb{1} - n_\downarrow) = b^\dagger b
\end{aligned}
\tag{14}
$$

with the physical subspace defined by $Q \equiv b^\dagger b + \sum_\sigma f_\sigma^\dagger f_\sigma = 1$. The physical meaning of the mapping is that the auxiliary fermions create single occupancies $|\sigma\rangle = f_\sigma^\dagger |\text{vac}\rangle$, while the bosons create the empty site $|0\rangle = b^\dagger |\text{vac}\rangle$, where $|\text{vac}\rangle$ is the vacuum in the extended Hilbert space. The effective Hamiltonian becomes

$$H_{\text{eff}} = \sum_\sigma \varepsilon_\sigma f_\sigma^\dagger f_\sigma + \sum_{p\sigma} \varepsilon_p c_{p\sigma\alpha}^\dagger c_{p\sigma\alpha} + \sum_{p\sigma} V_p (b f_\sigma^\dagger c_{p\sigma\alpha} + \text{H.c.}) \,, \tag{15}$$

and the effective jump operator reads

$$L_{\text{eff}} = \frac{2}{\gamma^{1/2}} b^\dagger \sum_{p\sigma} \sigma V_p f_{\bar{\sigma}} c_{p\sigma\alpha} \,, \tag{16}$$

corresponding to the Lindbladian dissipative action [8]:

$$\begin{aligned} S_d^{\text{eff}} = &- \mathrm{i}\frac{4\eta}{\gamma} \int \mathrm{d}t \sum_{\substack{p,p' \\ \sigma,\sigma'}} \sigma\sigma' V_p V_{p'} \Big[b_-(t)\bar{c}_{p\sigma-}(t)\bar{f}_{\bar{\sigma}-}(t)\bar{b}_+(t) f_{\bar{\sigma}'+}(t) c_{p'\sigma'+}(t) \\ &- \frac{1}{2} b_+(t)\bar{c}_{p\sigma+}(t)\bar{f}_{\bar{\sigma}+}(t)\bar{b}_+(t-0) f_{\bar{\sigma}'+}(t-0) c_{p'\sigma'+}(t-0) \\ &- \frac{1}{2} b_-(t)\bar{c}_{p\sigma-}(t)\bar{f}_{\bar{\sigma}-}(t)\bar{b}_-(t+0) f_{\bar{\sigma}'-}(t+0) c_{p'\sigma'-}(t+0) \Big] \,, \end{aligned} \tag{17}$$

where $t \pm 0$ indicates an infinitesimal shift of the times that is necessary to correctly calculate tadpole contributions to the self-energies.

The non-crossing approximation (NCA) amounts to a self-consistent perturbation theory (i.e. a skeleton expansion of the self-energy) in the impurity-lead hopping V_p truncated at the leading order [4, 7, 9–13], namely the second. The residual dissipation needs to be included at the same order. Since the dissipative vertex Eq. (17) is already of order V_p^2, we only need to include it at the mean-field (i.e. tadpole) level. The resulting approximation to the self-energy is then conserving [9, 11–13]. The Luttinger-Ward functional [14] truncated to the second order in the impurity-bath hopping is shown in Supplementary Figure 1, along with the relevant self-energies.

The only nontrivial aspect of the slave boson mapping is that the constraint $Q = 1$ has to be taken into account exactly. This can be done in a standard fashion [4, 9–13] by endowing the auxiliary particles with a fictitious chemical potential that is taken to infinity to extract the physical observables. Following Refs. [12, 13], we implement this constraint by discarding all terms that feature one or more lesser Green's function (bosonic or fermionic) in the retarded self-energy, and keeping at most one lesser Green's function in the expression of the lesser self-energy.

We are going to derive the equations describing the time evolution of the system prepared in a factorized state $\rho_0 = \chi_d \otimes \rho_l$ between the impurity χ_d and the leads ρ_d. For our purposes, the initial state of the impurity is completely characterized by the initial occupancy of the dot site $n_\sigma^0 = \langle d_\sigma^\dagger d_\sigma \rangle_\chi$, which coincides with the auxiliary fermion occupancy $\langle f_\sigma^\dagger f_\sigma \rangle = \langle d_\sigma^\dagger d_\sigma \rangle$ (and determines the initial boson occupancy

$\langle b^\dagger b\rangle_\chi = 1 - \sum_\sigma n^0_\sigma$ through the constraint). The leads are assumed to be prepared in their own equilibrium states $\rho_l = \bigotimes_\alpha \exp(-\beta H_\alpha)/Z_\alpha$, where $H_\alpha = \sum_{p\sigma\alpha}(\varepsilon_p - \mu_\alpha)c^\dagger_{p\sigma\alpha}c_{p\sigma\alpha}$ and $Z_\alpha = \mathrm{Tr}\exp(-\beta H_\alpha)$. The subsequent evolution can be thought as a quench in which the dot sites and the leads are initially disconnected (i.e. $V_p = 0$) and suddenly put into contact at time $t = 0$ by turning on the dot-leads hopping V_p. Hence, we will consider a time-dependent $V_p(t) = \theta(t)V_p$, which is necessary to set the correct limits in some integrals over time. The time dependence of the tunneling terms, i.e. the perturbation, makes all self energies $\Sigma_{f\sigma}(t,t')$, $\Pi(t,t')$ vanish whenever either of their time arguments is negative. Thus, we will always consider $t \geq 0$, $t' \geq 0$ in the following equations, and we will consequently omit the θ functions in front of the self-energies.

The contour-ordered NCA self-energies associated to the unitary part of the dynamics of the auxiliary particles f_σ and b are given by [4, 7, 9–13]

$$\begin{aligned}\Sigma_{f\sigma}(t,t') &= \mathrm{i}\boldsymbol{\Delta}_\sigma(t,t')B(t,t') \quad \text{for auxiliary fermions,}\\ \Pi(t,t') &= -\mathrm{i}\sum_\sigma \boldsymbol{\Delta}_\sigma(t',t)G_\sigma(t,t') \quad \text{for bosons}\end{aligned} \tag{18}$$

where $G_\sigma(t,t') \equiv -\mathrm{i}\left\langle \mathcal{T} f_\sigma(t) f^\dagger_\sigma(t')\right\rangle$ and $B(t,t') \equiv -\mathrm{i}\left\langle \mathcal{T} b(t) b^\dagger(t')\right\rangle$ are the contour-ordered Green's functions for the auxiliary fermions and the bosons, respectively ($\mathcal{T}$ is the contour-ordering symbol). The function $\boldsymbol{\Delta}_\sigma(t,t')$ is the local Green's function of the leads, namely $\boldsymbol{\Delta}_\sigma(t,t') \equiv \sum_{p,\alpha,p',\alpha'} V_p(t) g_{p\sigma\alpha,p'\sigma\alpha'}(t,t')V_p(t')$, where $g_{p\sigma\alpha,p'\sigma\alpha'}(t,t') \equiv -\mathrm{i}\left\langle \mathcal{T} c_{p\sigma\alpha}(t) c^\dagger_{p'\sigma\alpha'}(t')\right\rangle$. Applying Langreth's rules [15] and the projection onto $Q = 1$ we obtain the various components of the self-energies:

$$\begin{aligned}\Sigma^{\gtrless}_{f\sigma}(t,t') &= \mathrm{i}\Delta^{\gtrless}_\sigma(t-t')B^{\gtrless}(t,t')\ ,\\ \Sigma^{R,A}_{f\sigma}(t,t') &= \mathrm{i}\Delta^{>}_\sigma(t-t')B^{R,A}(t,t')\end{aligned} \tag{19}$$

and

$$\begin{aligned}\Pi^{\gtrless}(t,t') &= -\mathrm{i}\sum_\sigma \Delta^{\lessgtr}_\sigma(t'-t)G^{\gtrless}_\sigma(t,t')\ ,\\ \Pi^{R,A}(t,t') &= -\mathrm{i}\sum_\sigma \Delta^{<}_\sigma(t'-t)G^{R,A}_\sigma(t,t')\ .\end{aligned} \tag{20}$$

The projection on the physical subspace has two effects on the self-energies. The first is that some contributions are discarded, while the second is that the local Green's function $\boldsymbol{\Delta}_\sigma(t,t')$ has to be substituted by its *unperturbed* version $\Delta_\sigma(t,t')$. This occurs because any correction to $\Delta_\sigma(t,t')$ must contain at least one lesser Green's function of the fermions or the bosons [12, 13], contrary to the projection rule. This substitution is not an approximation, and it does not imply that the leads are not affected by the impurity. In fact, we will show in the section devoted to transport that the knowledge of the Green's functions of the auxiliary particles is sufficient to compute $g_{p\sigma\alpha,p'\sigma\alpha'}(t,t')$.

Since the unperturbed leads are assumed to be in thermodynamic equilibrium, we have [remembering $V_p(t) = \theta(t)V_p$] $\Delta_\sigma(t,t') \equiv \theta(t)\theta(t')\Delta_\sigma(t-t')$ and we can express

$\Delta_\sigma(t-t')$ through its Fourier transform $\Delta_\sigma(\omega) = \int \mathrm{d}t\, \mathrm{e}^{\mathrm{i}\omega^+(t-t')}\Delta_\sigma(t-t')$ (where $\omega^\pm \equiv \omega \pm \mathrm{i}0$ denotes an infinitesimal shift along the imaginary axis): $\Delta_\sigma(\omega) = \sum_\alpha \Delta_{\sigma\alpha}(\omega)$ where

$$\Delta^{R,A}_{\sigma\alpha}(\omega) = \int \frac{\mathrm{d}\varepsilon}{2\pi}\frac{\Gamma_\alpha(\varepsilon)}{\omega^\pm - \varepsilon} = \mathcal{P}\int \frac{\mathrm{d}\varepsilon}{2\pi}\frac{\Gamma_\alpha(\varepsilon)}{\omega - \varepsilon} \mp \frac{\mathrm{i}}{2}\Gamma_\alpha(\omega) \tag{21a}$$

$$\Delta^<_{\sigma\alpha}(\omega) = \mathrm{i}\Gamma_\alpha(\omega)F_\alpha(\omega) \tag{21b}$$

$$\Delta^>_{\sigma\alpha}(\omega) = -\mathrm{i}\Gamma_\alpha(\omega)[1 - F_\alpha(\omega)] \tag{21c}$$

We have introduced the level width function $\Gamma_\alpha(\omega) \equiv 2\pi \sum_p V^2_{p\alpha}\delta(\omega - \varepsilon_p)$ and the Fermi distribution $F_\alpha(\omega) \equiv \{\exp[\beta(\omega - \mu_\alpha)] + 1\}^{-1}$ of the lead α, with inverse temperature β [1]. Notice that we keep the label σ for the sake of generality, although we never consider the presence of magnetic fields or initially spin-unbalanced leads that could lead to an explicit spin dependence of Δ. Also, we temporarily keep the label α on Γ_α, although we will always consider two identical baths with $\Gamma_L = \Gamma_R$.

In our calculations, we assume zero temperature $\beta \to \infty$ and we take a flat density of states $\Gamma(\omega) = \Gamma\xi(\omega)$, with $\xi(\omega) = \theta(W - |\omega|)$, for which

$$\begin{aligned} \Delta^<_{\sigma\alpha}(t) &= -\frac{\Gamma}{2\pi}\frac{\mathrm{e}^{-\mathrm{i}\mu_\alpha t} - \mathrm{e}^{\mathrm{i}Wt}}{t} \\ \Delta^>_{\sigma\alpha}(t) &= \frac{\Gamma}{2\pi}\frac{\mathrm{e}^{-\mathrm{i}Wt} - \mathrm{e}^{-\mathrm{i}\mu_\alpha t}}{t} \end{aligned} . \tag{22}$$

The dissipative contributions to the retarded self-energies are

$$\begin{aligned} \Sigma^{\mathrm{diss},R}_{f\sigma}(t,t') &= -\mathrm{i}\frac{2}{\gamma}B^>(t,t)\boldsymbol{\Delta}^<_\sigma(t,t)\delta(t-t') \\ &\overset{\mathrm{proj.}}{\to} -\mathrm{i}\frac{2}{\gamma}[B^>(t,t) - B^<(t,t)]\Delta^<_\sigma(t,t)\delta(t-t') \\ &= -\frac{2}{\gamma}\Delta^<_\sigma(t,t)\delta(t-t') \equiv -\frac{\mathrm{i}}{2}\kappa_\sigma\delta(t-t') \\ \Pi^{\mathrm{diss},R}(t,t') &= -\frac{2\mathrm{i}}{\gamma}\sum_\sigma \boldsymbol{\Delta}^<_\sigma(t,t)G^<_{\bar\sigma}(t,t)\delta(t-t') \overset{\mathrm{proj.}}{\to} 0 \end{aligned} \tag{23}$$

where $\overset{\mathrm{proj.}}{\to}$ stands for the projection procedure of discarding all lesser fermionic or bosonic Green's functions, mentioned in the previous paragraphs. In the above Equations we introduced the effective dissipation rate

$$\kappa_\sigma \equiv -\mathrm{i}\frac{4}{\gamma}\Delta^<_{\bar\sigma}(t,t) = \sum_\alpha \frac{2\Gamma_\alpha}{\pi\gamma}\int_{-\infty}^{\infty}\mathrm{d}\omega\,\xi(\omega)F_\alpha(\omega) \ , \tag{24}$$

[1]Since we are not interested in thermal transport, we assume that both baths have the same temperature.

where $\xi(\omega)$ is the cutoff function defined by $\Gamma_\alpha(\omega) = \Gamma_\alpha \xi(\omega)$. For a flat level width function $\xi(\omega) = \theta(W - |\omega|)$ we have

$$\kappa_\sigma = 2 \sum_\alpha \frac{\Gamma_\alpha}{\pi \gamma} (\mu_\alpha + W) \ , \tag{25}$$

while for a general shape $\xi(\omega)$ we can estimate $\kappa \sim \Gamma/\gamma \cdot W$. Thus, the effective loss rate depends on the full band-shape of the leads, and grows with the bandwidth.

The lesser self-energies read

$$\begin{aligned}
&\Sigma^{\mathrm{diss},<}_{f\sigma}(t,t') = 0 \\
&\Pi^{\mathrm{diss},<}(t,t') = \frac{4\mathrm{i}}{\gamma} \boldsymbol{\Delta}^<_\sigma(t,t) G^<_{\bar\sigma}(t,t)\delta(t-t') \\
&\qquad \overset{\text{proj.}}{\to} \frac{4\mathrm{i}}{\gamma} \sum_\sigma \Delta^<_\sigma(t,t) G^<_{\bar\sigma}(t,t)\delta(t-t') = -\sum_\sigma \kappa_\sigma G^<_\sigma(t,t)\delta(t-t')
\end{aligned} \tag{26}$$

As anticipated, the leading order self-energies from the effective interaction are mean-field-like corrections, because they are local in time.

Dyson equations

Using the self-energies written above, we can write down the Dyson equations determining the dynamics of the retarded Green's functions

$$\begin{aligned}
(\mathrm{i}\partial_t - \varepsilon_\sigma) G^R_\sigma(t,t') &= \delta(t-t') - \frac{\mathrm{i}}{2}\kappa_\sigma G^R_\sigma(t,t') + \mathrm{i} \int_{t'}^t \mathrm{d}\bar t \, \Delta^>_\sigma(t-\bar t) B^R(t,\bar t) G^R_\sigma(\bar t, t') \ , \\
(-\mathrm{i}\partial_{t'} - \varepsilon_\sigma) G^R_\sigma(t,t') &= \delta(t-t') - \frac{\mathrm{i}}{2}\kappa_\sigma G^R_\sigma(t,t') + \mathrm{i} \int_{t'}^t \mathrm{d}\bar t \, G^R_\sigma(t,\bar t) \Delta^>_\sigma(\bar t - t') B^R(\bar t, t')
\end{aligned} \tag{27}$$

for the auxiliary fermions, and

$$\begin{aligned}
\mathrm{i}\partial_t B^R(t,t') &= \delta(t-t') - \mathrm{i} \sum_\sigma \int_{t'}^t \mathrm{d}\bar t \, \Delta^<_\sigma(\bar t - t) G^R_\sigma(t,\bar t) B^R(\bar t, t') \ , \\
-\mathrm{i}\partial_{t'} B^R(t,t') &= \delta(t-t') - \mathrm{i} \sum_\sigma \int_{t'}^t \mathrm{d}\bar t \, B^R(t,\bar t) \Delta^<_\sigma(t' - \bar t) G^R_\sigma(\bar t, t')
\end{aligned} \tag{28}$$

for the bosons. The advanced Green's functions can be obtained as $G^A(t,t') = [G^R(t',t)]^*$, with $G = G_\sigma$ or B. The boundary conditions for the Dyson equations are $G^R_\sigma(t+0,t) = B^R(t+0,t) = -\mathrm{i}$, which simply enforces the (anti-)commutation relations. A peculiar behavior of the Dyson equations in the NCA is that they only involve retarded functions, which greatly simplifies their numerical solution. In particular, the retarded Green's function that are obtained are time-translational invariant: $G^R_\sigma(t,t') \equiv G^R_\sigma(t-t')$, $B^R(t,t') \equiv B^R(t-t')$. For $\kappa = 0$ we obtain the same solution as in equilibrium. If this behavior might seem unphysical, let us recall that it regards the

dynamics of two unphysical, auxiliary particles f_σ and b of which the physical fermion $d_\sigma = b^\dagger f_\sigma$ [cf. Eqs. (14)] is “composed”. Indeed, the object that we aim to compute is the physical Green’s function for the d_σ operators, which in the language of the slave boson representation is a four-point vertex. For this object, the time evolution of the retarded and lesser components are intertwined.

Kinetic equations

The lesser components of the auxiliary fermion Green’s functions are determined by

$$
\begin{aligned}
(\mathrm{i}\partial_t - \varepsilon_\sigma) G^<_\sigma(t,t') &= -\frac{\mathrm{i}}{2}\kappa_\sigma G^<_\sigma(t,t') + \mathrm{i}[(\Delta^>_\sigma B^R) * G^<_\sigma](t,t') + \mathrm{i}[\Delta^<_\sigma B^< * G^A_\sigma](t,t') \ , \\
(-\mathrm{i}\partial_{t'} - \varepsilon_\sigma) G^<_\sigma(t,t') &= +\frac{\mathrm{i}}{2}\kappa_\sigma G^<_\sigma(t,t') + [G^R_\sigma * \mathrm{i}\Delta^<_\sigma B^<](t,t') + [G^<_\sigma * \mathrm{i}\Delta^>_\sigma B^A](t,t') \ .
\end{aligned}
\tag{29}
$$

The above equations have been written in a compact notation that treats the Green’s functions as matrices in the two time indices. We have introduced two different “products” for the Green’s functions: a direct product $(AB)(t,t') = A(t,t')B(t,t')$ and the time convolution (i.e. the matrix product) $(A*B)(t,t') \equiv \int \mathrm{d}\bar{t}\, A(t,\bar{t})B(\bar{t},t')$. The time evolution of the diagonal component reads

$$
\begin{aligned}
\mathrm{i}\mathrm{d}_t G^<_\sigma(t,t) &= [\mathrm{i}\partial_t G^<_\sigma(t,t') + \mathrm{i}\partial_{t'} G^<_\sigma(t,t')]\big|_{t'=t} = \\
&= -\mathrm{i}\kappa_\sigma G^<_\sigma(t,t) \\
&\quad + 2\,\mathrm{Re}\left\{\mathrm{i}\int_0^t \mathrm{d}\bar{t}\left[\Delta^<_\sigma(t-\bar{t})B^<(t,\bar{t})G^A_\sigma(\bar{t},t) + \Delta^>_\sigma(t-\bar{t})B^R(t,\bar{t})G^<_\sigma(\bar{t},t)\right]\right\}
\end{aligned}
\tag{30}
$$

The boundary conditions are simply the initial dot populations: $G_\sigma(0,0) = \mathrm{i}n_{d\sigma}(0)$.

For the bosons we similarly obtain

$$
\begin{aligned}
\mathrm{i}\partial_t B^<(t,t') &= -\sum_\sigma \kappa_\sigma G^<_\sigma(t,t) B^A(t-t') \\
&\quad - \mathrm{i}\sum_\sigma [(\Delta^<_\sigma)^T G^R_\sigma * B^<](t,t') - \mathrm{i}\sum_\sigma [(\Delta^>_\sigma)^T G^<_\sigma * B^A](t,t') \ , \\
-\mathrm{i}\partial_{t'} B^<(t,t') &= -\sum_\sigma \kappa_\sigma G^<_\sigma(t,t) B^R(t-t') \\
&\quad - [B^R * \mathrm{i}\sum_\sigma (\Delta^>_\sigma)^T G^<_\sigma](t,t') - [B^< * \mathrm{i}\sum_\sigma (\Delta^<_\sigma)^T G^A_\sigma](t,t')
\end{aligned}
\tag{31}
$$

where we employ the notation $(\Delta^T)(t,t') = \Delta(t',t)$. To find the equation determining the evolution of the diagonal component one uses $\lim_{t'\to t}[B^A(t,t') - B^R(t,t')] =$

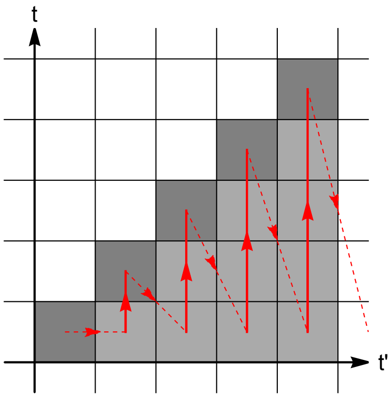

Supplementary Figure 2: Sketch of the steps for the numerical integration for the determination of the lesser Green's functions. The first square on the diagonal is the initial condition $G^<_\sigma(0,0) = \mathrm{i}n^0_\sigma$, $B^<(0,0) = 1 - \sum_\sigma n^0_\sigma$, and the subsequent integrations proceed along the columns, from $t' = 0$ to $t' = t$ (thick red arrows). The steps corresponding to the light gray squares make use of Eqs. (29) and (31), while the diagonal elements in darker gray employ Eqs. (30) and (32).

$\lim_{t'\to t}[B^<(t,t') - B^>(t,t')] = -\mathrm{i}\big[b^\dagger, b\big] = \mathrm{i}$ and finds

$$\mathrm{i}\mathrm{d}_t B^<(t,t) = -\mathrm{i}\sum_\sigma \kappa_\sigma G^<_\sigma(t,t) + 2\,\mathrm{Re}\left\{\mathrm{i}\sum_\sigma \int_0^t \mathrm{d}\bar{t}\left[\Delta^<_\sigma(t-\bar{t})B^<(t,\bar{t})G^A_\sigma(\bar{t},t) + \Delta^>_\sigma(t-\bar{t})B^R(t,\bar{t})G^<_\sigma(\bar{t},t)\right]\right\}. \tag{32}$$

It is easy to observe that the constraint is explicitly conserved: $\mathrm{d}_t Q(t) = \mathrm{i}\mathrm{d}_t B^<(t,t) - \sum_\sigma \mathrm{i}\mathrm{d}_t G^<_\sigma(t,t) = 0$. So, if we take the appropriate initial condition $B(0,0) = -\mathrm{i}[1 - \sum_\sigma n_{d\sigma}(0)]$, we will always keep $Q = 1$ during the dynamics, within the numerical accuracy of our computations.

Numerical implementation

We solve the Equations above using the simple algorithm described in Ref. [13]. Conceptually, we have equations in the form $\frac{\mathrm{d}}{\mathrm{d}t}f(t) = K[f](t)$, where the right-hand side depends functionally on f. We first convert the equation to an integral one to obtain one integration step: $\int_t^{t+\delta t}\mathrm{d}t'\,\frac{\mathrm{d}}{\mathrm{d}t'}f(t') = f(t+\delta t) - f(t) = \int_t^{t+\delta t}\mathrm{d}t'\,K[f](t')$. The integrals on the right-hand side (i.e. the one for the time step and the one implied in K) are computed by any quadrature rule. In our case, the simple trapezoidal rule with equally spaced points suffices. This choice leads to clearer equations, without sacrificing too much the accuracy (at the cost of needing a δt which cannot be too large). A key point is that the update rule for $f(t+\delta t)$ is implicit, since $f(t+\delta t)$ appears also in the discretization of the right-hand side. This makes the integration numerically stable. A nice feature of the implementation of Ref. [13] is that Q is exactly

conserved also in the discretized dynamics, which implies that the deviations from 1 will be comparable by machine precision $\sim 10^{-14\div 15}$.

The Dyson Eqs. (27) and (28) are integrated first, since they do not depend on the lesser functions. The equations for the fermionic and bosonic Green's functions are coupled, though, and need to be solved together. We only integrate the forward equations $\mathrm{i}\partial_t G_\sigma(t-t') = \ldots$, etc., and we take advantage of the time-translational invariance of the retarded Green's functions. The retarded functions thus obtained are then used to compute the lesser functions. The causal properties of the equations implies that we need to follow the path depicted in Supplementary Figure 2 on the discretized time grid (t, t'). Since lesser Green's functions are anti-Hermitian matrices in the time indices [15, 16], we compute only their values for $t \leq t'$.

In most of our calculations we set the time step to $\delta t = 0.5W^{-1}$. We have verified that doubling or halving this value leads to differences of at most a few per mil in Green's functions and currents. The value of the observables after the initial transient, i.e. for times larger than a few Γ^{-1}, are less susceptible to changes of δt—a possible manifestation of the dynamically attractive nature of the local stationary state.

Physical Green's function

In the subspace with no double occupancies $d_\sigma = X_{0\sigma} = b^\dagger f_\sigma$. Hence

$$G_{d\sigma}(t,t') = -\mathrm{i}\left\langle \mathcal{T}_{\mathcal{C}} d_\sigma(t) d^\dagger_\sigma(t')\right\rangle = -\mathrm{i}\left\langle \mathcal{T}_{\mathcal{C}} f_\sigma(t) b^\dagger(t) b(t') f^\dagger_\sigma(t')\right\rangle \ . \tag{33}$$

Within the non-crossing approximation [4, 6, 7, 9–13] one simply decouples the fermions from the bosons (i.e. one ignores vertices):

$$G_{d\sigma}(t,t') \approx \mathrm{i} G_\sigma(t,t') B(t',t) \ . \tag{34}$$

This approximation can be obtained as the leading term in a large-N expansion in the number of flavors of fermions (with the rescaling $V_p \to V_p/N^{1/2}$). While the auxiliary particles' Green functions are approximated with a good accuracy by the NCA equations derived above, the neglect of vertices in the physical Green's function does introduce some spurious effects [17, 18]. Nevertheless, it is known to provide the correct qualitative properties of the Kondo effect, with quantitative discrepancies of the order of 15% for observables such as the zero-bias conductance [10]. We obtain the lesser function as follows:

$$\begin{aligned} G^<_{d\sigma}(t,t') &\approx \mathrm{i} G^<_\sigma(t,t') B^>(t',t) \\ &\overset{\text{proj.}}{\to} \mathrm{i} G^<_\sigma(t,t')[B^>(t',t) - B^<(t',t)] = G^<_\sigma(t,t') b(t',t) \ , \end{aligned} \tag{35}$$

where the first approximate equality is the NCA, while the second equality is the projection onto the physical subspace $Q = 1$. We have introduced the functions

$$\begin{aligned} g_\sigma(t,t') &\equiv \mathrm{i}[G^>_\sigma(t,t') - G^<_\sigma(t,t')] \\ b(t,t') &\equiv \mathrm{i}[B^>(t,t') - B^<(t,t')] \end{aligned} \tag{36}$$

so that

$$\begin{aligned} G^R_\sigma(t,t') &= -\mathrm{i}\theta(t-t')g_\sigma(t-t') \\ G^A_\sigma(t,t') &= +\mathrm{i}\theta(t'-t)g_\sigma(t,t') \end{aligned} \tag{37}$$

and analogously for the bosons. In particular, $b(t,t) = 1$, so $\langle d_\sigma(t)^\dagger d_\sigma(t)\rangle \equiv -\mathrm{i}G^<_{d\sigma}(t,t) = -\mathrm{i}G^<_\sigma(t,t) = \langle f_\sigma(t)^\dagger f_\sigma(t)\rangle$, where the equality $G^<_{d\sigma}(t,t) = G^<_\sigma(t,t)$ is exact. Similarly,

$$\begin{aligned} G^>_{d\sigma}(t,t') &\approx \mathrm{i}G^>_\sigma(t,t')B^<(t',t) \\ &\overset{\text{proj.}}{\to} \mathrm{i}[G^>_\sigma(t,t') - G^<_\sigma(t,t')]B^<(t',t) = g_\sigma(t,t')B^<(t',t)\ , \end{aligned} \tag{38}$$

and finally

$$\begin{aligned} G^R_{d\sigma}(t,t') &\equiv \theta(t-t')\big[G^>_{d\sigma}(t,t') - G^<_{d\sigma}(t,t')\big] \\ &= \mathrm{i}\big[G^R_\sigma(t,t')B^<(t',t) + G^<_\sigma(t,t')B^A(t',t)\big] \end{aligned} \tag{39}$$

At first sight, there appears to be a problem with the anticommutation relations $\{d_\sigma, d^\dagger_\sigma\} = \mathbb{1}$: $G^>_{d\sigma}(t,t) = B^<(t,t) = -\mathrm{i} + \sum_\sigma G^<_\sigma(t,t) \neq -\mathrm{i} + G^<_\sigma(t,t)$, therefore

$$G^R_{d\sigma}(t+0,t) = B^<(t,t) - G^<_\sigma(t,t) = -\mathrm{i}(1 - G^<_{-\sigma}(t,t)) \neq -\mathrm{i}\{d_\sigma, d^\dagger_\sigma\} = -\mathrm{i} \tag{40}$$

This is not a shortcoming of the NCA, but rather of the Hilbert space truncation $d_\sigma \to X_{0\sigma}$. Indeed, $-\mathrm{i}\{X_{0\sigma}, X_{\sigma 0}\} = -\mathrm{i}(X_{00} + X_{\sigma\sigma}) \neq \mathbb{1}$. This modified anticommutation relation means that the spectral function will not integrate to 1, but rather to $\mathrm{i}(B^<(t,t) - G^<_\sigma(t,t)) = 1 - n_{-\sigma}(t)$. The missing spectral weight is that of double occupancies, which would appear as a very broad and low peak at $\omega = \varepsilon_d$, with width $\sim \gamma$ and height $\sim \gamma^{-1}$.

In the main text, we show the dot spectral function in the local stationary state. This function is defined as $A_\sigma(\omega,t) = -\operatorname{Im} G^R_{d\sigma}(\omega,t)/\pi$, where $G^R_{d\sigma}(\omega,t) \equiv \int_0^\infty \mathrm{d}\tau\, \mathrm{e}^{\mathrm{i}\omega\tau} G^R_{d\sigma}(t+\tau,t)$, choosing $t \gtrsim 5\Gamma_T^{-1}$ such that the $A_\sigma(\omega,t)$ has already saturated to its late-time value.

Transport

As usual in impurity problems [15], the Green's function of the leads $g_{p\sigma\alpha,p'\sigma\alpha'}(t,t')$ is determined by a T-matrix equation

$$\hat{g} = \hat{g}_0 + \hat{g}_0 * \hat{P} * \hat{g}_0\ , \tag{41}$$

where the hat indicates a matrix in p, σ, α and times, and with the convolution symbol $*$ representing a matrix multiplication in all of the above indices. The T-matrix $\hat{P}$ on the contour can be obtained by deriving the Luttinger-Ward functional (Supplementary Figure 1) and reads

$$P_{p\sigma\alpha,p'\sigma'\alpha'}(t,t') = V_{p\alpha}V_{p'\alpha'}\delta_{\sigma\sigma'}\Big[\mathrm{i}G_\sigma(t,t')B(t',t) + \frac{4}{\gamma}G^<_\sigma(t,t)\delta(t-t')\Big]\ . \tag{42}$$

When applying Langreth's rules to determine the various components of $\hat{P}$ we must apply the projection [12, 13] on the physical subspace $Q = 1$. In this case there is a subtlety: the rule of keeping at most one auxiliary bosonic or fermionic lesser function applies only if the projected quantity vanishes in the unphysical $Q = 0$ space. This is not the case for $\hat{g}$, since $\hat{g}_{Q=0} = \hat{g}_0 \neq 0$. Therefore, the projection must be done on $\hat{g} - \hat{g}_0$, and we obtain that $P = \mathcal{O}(G^<_\sigma, B^<)$ for all components. We can write the results in terms of a new function $Q_\sigma(t,t')$ (not to be confused with the charge Q) defined by

$$P_{p\sigma\alpha,p'\sigma'\alpha'}(t,t') = V_{p\alpha}V_{p'\alpha'}\delta_{\sigma\sigma'}Q_\sigma(t,t') \; . \tag{43}$$

Then, within the NCA we have $Q^{R,<}_\sigma(t,t') = G^{R,<}_{d\sigma}(t,t') + Q^{\mathrm{diss},R,<}_\sigma(t)\delta(t-t')$, with

$$\begin{aligned} Q^{\mathrm{diss},R}_\sigma(t) &= -\frac{2}{\gamma}G^<_{\bar\sigma}(t,t) = -\frac{2\mathrm{i}}{\gamma}n_{\bar\sigma}(t) \\ Q^{\mathrm{diss},<}_\sigma(t) &= 0 \end{aligned} \tag{44}$$

The current leaving reservoir α is defined as

$$I_\alpha \equiv -\frac{\mathrm{d}}{\mathrm{d}t}\sum_{p\sigma}\langle c^\dagger_{p\sigma\alpha}c_{p\sigma\alpha}\rangle_t = -\frac{\mathrm{d}}{\mathrm{d}t}\sum_{p\sigma}g^<_{p\sigma\alpha,p\sigma\alpha}(t,t) \; . \tag{45}$$

With the help of the appropriate T-matrix equation $\hat{g}^< = \hat{g}^<_0 + \hat{g}^<_0 * \hat{P}^A * \hat{g}^A_0 + \hat{g}^R_0 * \hat{P}^< * \hat{g}^A_0 + \hat{g}^R_0 * \hat{P}^R * \hat{g}^<_0$ and the NCA expression for $\hat{P}$ and $\hat{Q}$ we find

$$\begin{aligned} I_\alpha(t) =& 2\,\mathrm{Re}\int_0^t \mathrm{d}\bar{t}\sum_\sigma\left[Q^R_\sigma(t,\bar{t})\Delta^<_{\sigma\alpha}(\bar{t}-t) + Q^<_\sigma(t,\bar{t})\Delta^A_{\sigma\alpha}(\bar{t}-t)\right] = \\ =& 2\,\mathrm{Re}\int_0^t \mathrm{d}\bar{t}\sum_\sigma\left[G^R_{d\sigma}(t,\bar{t})\Delta^<_{\sigma\alpha}(\bar{t}-t) + G^<_{d\sigma}(t,\bar{t})\Delta^A_{\sigma\alpha}(\bar{t}-t)\right] \\ &+ \frac{4}{\gamma}\sum_\sigma n_{\bar\sigma}(t)(-\mathrm{i})\Delta^<_{\sigma\alpha}(0) \; . \end{aligned} \tag{46}$$

The first term in the second line of the above Equation is the usual starting point of the Meir-Wingreen formula [15, 19], whereas the second term represents a new, dissipative contribution. We will show later that this latter contribution is related to the loss of particles from the system—a conclusion that can be anticipated by its proportionality to the dot occupancy. The loss term is proportional to the Green's function of the leads evaluated at coinciding times, $-\mathrm{i}\Delta^<_{\sigma\alpha}(0)$, and is therefore non-universal, in the sense that it is sensitive to the full band shape of the leads, $\xi(\omega)$. For an infinite bandwidth W, this term would diverge [2].

We can then easily derive the conductance at zero bias, defined as $g(0,t) \equiv \lim_{\Delta\mu\to 0} \mathrm{d}I(t)/\mathrm{d}\Delta\mu$, where the transport current is $I(t) \equiv (I_L(t) - I_R(t))/2$, assuming

[2]This limit cannot be taken, however, since the derivation of the effective Hamiltonian assumes $\gamma \gg W$.

that the left reservoir is at a higher chemical potential, $\mu_{L,R} = \mu \pm \Delta\mu/2$. Using

$$\frac{\mathrm{d}}{\mathrm{d}\Delta\mu} F_{L,R}(\omega) = \frac{\mathrm{d}}{\mathrm{d}\Delta\mu} F\Big[\omega - \big(\mu \pm \tfrac{\Delta\mu}{2}\big)\Big] = \mp\frac{1}{2} F'(\omega - \mu_\alpha) \tag{47}$$

in the expression for $\Delta^<_{\sigma\alpha}$ (with the prime F' indicating the derivative), we obtain at zero temperature

$$g(0,t) = -\frac{\Gamma(\mu)}{2} \sum_\sigma \frac{1}{\pi} \,\mathrm{Im} \int_0^t \mathrm{d}\bar{t}\, \mathrm{e}^{\mathrm{i}\mu\bar{t}} G^R_{d\sigma}(t, t-\bar{t}) + \frac{\Gamma(\mu)}{\pi\gamma} n_d(t) \ , \tag{48}$$

which is Eq. (2) quoted in the main text. As noted there, the first term directly probes the spectral function at the chemical potential, and at later times converges to the usual stationary formula [15, 19] $g_\infty(0) = 2^{-1}\Gamma(\mu)\sum_\sigma A_\sigma(\mu)$. Thus, the zero-bias conductance allows to detect directly the presence of the Kondo peak. Moreover, it is interesting to notice that the first term of Eq. (48) only probes the spectral function at the chemical potential of the leads, thus displaying universal behavior in the usual Hamiltonian case. On the other hand, the correction represented by the second term explicitly depends on the dot population $n_d(t)$, which is non-universal (i.e. explicitly dependent on the cutoff W and band shape $\xi(\omega)$). On the other hand, it does not diverge for an infinite bandwidth, unlike the individual currents $I_\alpha(t)$. Indeed, the "loss" terms of the currents, i.e. the last term of (46), partially cancel out in the transported current even for a finite bias:

$$\begin{aligned} \frac{2}{\gamma} \sum_\sigma n_{\bar\sigma}(t)(-\mathrm{i})[\Delta^<_{\sigma L}(0) - \Delta^<_{\sigma R}(0)] &= \frac{2}{\gamma} n_d(t) \int \frac{\mathrm{d}\omega}{2\pi} \Gamma(\omega) \big[F_L(\omega) - F_R(\omega)\big] \\ &= \frac{2}{\gamma} n_d(t) \int_{\mu_R}^{\mu_L} \frac{\mathrm{d}\omega}{2\pi} \Gamma(\omega) \ , \end{aligned} \tag{49}$$

where the last equality applies to zero temperature. In words, the losses contribute to the transport current only through the lead fermions present between the two chemical potentials. We need to remark that the loss correction to the zero-bias conductance is tiny with respect to the transport term (i.e. the first one of Eq. (48)): while the latter is of order $10^{-1\div 0}$, the former is at most (for the maximal $n_d(t) = 1$) $\Gamma/(\pi\gamma)$, which in our calculation is always less than $3 \cdot 10^{-3}$. Thus, almost all of the effect of the residual dissipation affects the conductance through the impurity spectral function, and in particular the decrease of the height of the Kondo peak for decreasing dissipation rate.

It is interesting to compare the behavior of the transported current with the current of particles lost from the system, $I_{\mathrm{loss}}(t) = -\,\mathrm{d}[N_R(t) + N_L(t) + n_d(t)]/\mathrm{d}t = I_R(t) + I_L(t) - \mathrm{d}n_d(t)/\mathrm{d}t$. Using the expressions (46), (30) and (39) we find

$$I_{\mathrm{loss}}(t) = \frac{4}{\gamma} \sum_\sigma n_{\bar\sigma}(t)(-\mathrm{i})\Delta^<_\sigma(0) = 2\sum_\sigma \kappa_\sigma n_\sigma(t) \ . \tag{50}$$

As a check, we notice that in the limit $\gamma \to +\infty$ κ_σ vanishes and we recover the statement of conservation of the number of particles: $I_{\text{loss}} = 0$. In general, after a transient of a few Γ^{-1} the dot population saturates, and for a finite γ the loss current saturates to $I_{\text{loss}}(t) \to I_{\text{loss}}^{\infty} = 2\sum_\sigma \kappa_\sigma n_\sigma^\infty$. As it could be expected on classical grounds, the stationary loss current is proportional to the dot population and to the density of leads fermions at the dot site, since $\kappa_\sigma \propto \Delta_\sigma^{<}(0)$. The factor of 2 accounts for the fact that each loss event entails the disappearance of two particles, one from the dot and one from the leads. In the large-γ regime that we are analyzing, the losses are suppressed by an explicit factor of γ (coming from κ_σ), besides of the slow increase of n_d^∞ with γ (see later). As we have already remarked, the dot population does not show universal behavior, hence the loss current does not provide a good probe of the Kondo effect emerging at large dissipation.

We can use $I_{\text{loss}}(t)$ to estimate the double occupancies $\langle d_\uparrow^\dagger d_\uparrow d_\downarrow^\dagger d_\downarrow \rangle$ that are still present in the Kondo regime. We compute $I_{\text{loss}}(t)$ in the full theory:

$$I_{\text{loss}}(t) = -\frac{\mathrm{d}}{\mathrm{d}t}\langle N_{\text{tot}}\rangle = -\frac{\gamma}{2}\left\langle \left[L^\dagger, N_{\text{tot}}\right]L + L^\dagger[L, N_{\text{tot}}]\right\rangle$$

where we took into account that the Hamiltonian part of the dynamics conserves the total number of fermions $N_{\text{tot}} = n_d + \sum_\alpha N_\alpha$. Using $[N_{\text{tot}}, L] = [n_d, L] = -2L$, we obtain:

$$I_{\text{loss}}(t) = 2\gamma \left\langle L^\dagger L \right\rangle(t) = 2\gamma \left\langle d_\uparrow^\dagger d_\uparrow d_\downarrow^\dagger d_\downarrow \right\rangle(t) \ . \tag{51}$$

Hence, the number of double occupancies is

$$\left\langle d_\uparrow^\dagger d_\uparrow d_\downarrow^\dagger d_\downarrow \right\rangle(t) = \frac{I_{\text{loss}}(t)}{2\gamma} = \frac{1}{\gamma}\sum_\sigma \kappa_\sigma n_\sigma(t) \ , \tag{52}$$

where in the last equality we used Eq. (50) from the effective model at large γ—we are assuming that the results of the full theory should converge smoothly to those of the effective one in the regime of validity of the latter. Recalling that $\kappa_\sigma \sim \mathcal{O}(\Gamma_T W/\gamma)$ and that $n_d(t)$ has only a weak dependence on γ, we estimate that in the Kondo regime $\langle d_\uparrow^\dagger d_\uparrow d_\downarrow^\dagger d_\downarrow \rangle(t) \sim \Gamma_T W/\gamma^2$ is suppressed as γ^{-2}.

Supplementary Note 3: Additional data

In this Section, we provide additional data on the relation between the loss rate γ and the presence of typical signatures of the Kondo effect.

In Supplementary Figure 3 we show the long-time nonlinear conductance $g_\infty = \lim_{t\to\infty} I(t)/\Delta\mu$ as a function of the ratio $\Delta\mu/T_K$. A clear signature of the Kondo effect for $\gamma \to +\infty$ (continuous lines) is that the curves from different dot energies ε_d collapse on a universal curve (except those in the mixed-valence regime $\varepsilon_d \gtrsim -\Gamma_T$) [2, 4, 10, 10, 11]. This scaling collapse signals the presence of T_K as the only energy scale governing the low-energy transport properties, and can be used to detect the Kondo effect. In the Figure, dashed and dotted lines refer to the finite-dissipation cases of $\gamma = 10W$ and $\gamma = W$, respectively. We observe that in this regime the scaling

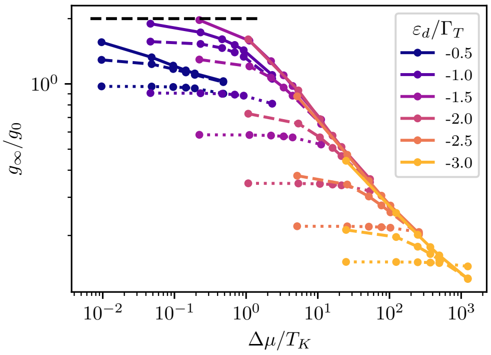

Supplementary Figure 3: Loss of scaling collapse in the nonlinear conductance in presence of a finite dissipation. The data are for $\Gamma_T = 0.02W$, $T_K = (\Gamma_T W/2)^{1/2}\exp(\pi\varepsilon_d/\Gamma_T)$. The full curves are for the AIM at $\gamma \to +\infty$, the dashed curves are for $\gamma = 10W$ and the dotted curves are for $\gamma = W$. The black dashed line marks the maximal conductance value $2g_0$. Decreasing γ, besides decreasing the conductance itself, spoils the data collapse as a function of $\Delta\mu/T_K$.

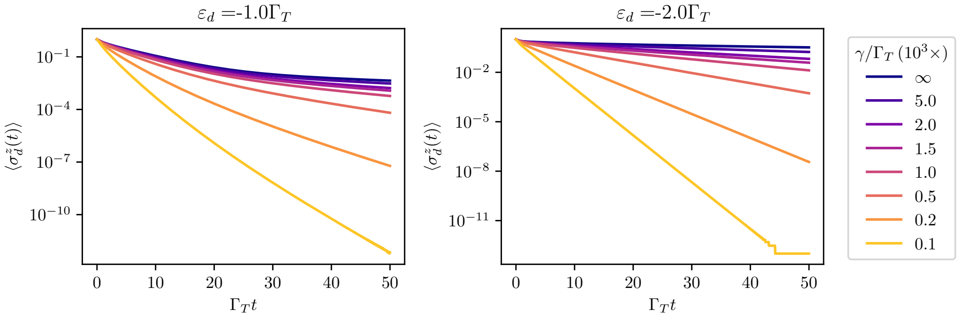

Supplementary Figure 4: Spin decay in real time for a dot in the mixed-valence regime (left) and in the Kondo regime (right). In both scenarios we can observe how the decay is suppressed by an increasing loss rate γ, thus signaling the regime of strong correlations. Both plots are for $\Gamma_T = 10^{-2}W$. The discrete steps in the $\gamma = 100\Gamma_T$ curve of the right plot are caused by the reaching of machine precision $\langle\sigma_d^z(t)\rangle \sim 10^{-13}$.

collapse is lost, since the new competing energy scale κ_σ emerges. Especially for the smaller $\gamma = W$, the conductance becomes scarcely dependent on the bias $\Delta\mu$. From Figure 1b in the main text, this mild dependence can be attributed to the complete loss of the Kondo peak in the spectral function, meaning that the dot behaves simply as a noninteracting dot, which has its maximal sensitivity to the bias only for $\mu \approx \varepsilon_d$.

In Supplementary Figure 4 we show the typical appearance of the spin decay in the mixed-valence regime $\varepsilon_d = -\Gamma_T$ (left) and in the Kondo regime $\varepsilon_d = -2\Gamma_T$ (right). While for $|\varepsilon_d| \lesssim \Gamma_T$ the decay is slower than exponential, for deeper dot levels a purely exponential form is recovered. Regardless of the form of the decay, the effect of the

two-body loss is to suppress the equilibration of the impurity spin, down to quite a small value. This suppression signals the crossover to the strongly dissipated regime in which the physics is dominated by the Kondo resonance (which corresponds precisely to a long-lived spin state [1]). The curves in Figure 1c in the main text are obtained via a linear fit of log $\langle \sigma_d^z(t) \rangle$ versus time, excluding the initial data for $t \leq 10\Gamma_T^{-1}$. According to the literature [20–22], the exponential decay rate of $\langle \sigma_d^z(t) \rangle$ in the Kondo regime should be proportional to the Kondo temperature—namely, it should scale exponentially with the ratio ε_d/Γ_T with a slope of π. While in Figure 1c in the main text we do observe an exponential scaling, we find that the slope is smaller, being close to 0.56π. A similar behavior is observed with the variational method in the companion paper [23].

Supplementary Note 4: Discussion of the many-sites setup

In this Section, we provide a detailed discussion of the setup in which the dot is composed of more than one site, and on the conditions for the mapping of this dissipative model to a higher-spin Kondo model.

We consider the Lindblad master equation for a composite dot made of ℓ_d sites, each one subject to a two-body decay rate of γ_j:

$$\frac{\mathrm{d}}{\mathrm{d}t}\rho(t) = -\mathrm{i}[H, \rho(t)] + \sum_{j \in \mathrm{dot}} \gamma_j \left(L_j \rho(t) L_j^\dagger - \frac{1}{2}\{L_j^\dagger L_j, \rho(t)\} \right) , \tag{53}$$

with jump operators $L_j \equiv d_{j\downarrow} d_{j\uparrow}$ (the operator $d_{j\sigma}$ annihilates a fermion with spin σ at the dissipative site j) and a Hamiltonian having the usual form $H = H_{\mathrm{dot}} + H_{\mathrm{leads}} + H_{\mathrm{tun}}$, with

$$\begin{aligned} H_{\mathrm{dot}} &= \sum_{i,j \in \mathrm{dot}, \sigma} h_{ij} d_{i\sigma}^\dagger d_{j\sigma} \\ H_{\mathrm{leads}} &= \sum_{p\sigma\alpha} \varepsilon_{p\alpha} c_{p\sigma\alpha}^\dagger c_{p\sigma\alpha} \\ H_{\mathrm{tun}} &= \sum_{pj\alpha\sigma} (V_{j,p\alpha} d_{j\sigma}^\dagger c_{p\sigma\alpha} + \mathrm{H.c.}) \end{aligned} , \tag{54}$$

where now we do not need to specify the Hermitian matrix h_{ij}, the bath energies $\varepsilon_{p\alpha}$ and the tunneling matrices $V_{j,p\alpha}$. The goal of the next paragraphs is to identify the dark states of the isolated dot, and this task does not rely on any specific form of the various parameters h_{ij}, $\varepsilon_{p\alpha}$, $V_{j,p\alpha}$. These dark states would then form the slow subspace (i.e. protected from dissipation in a suitable $\gamma_j \to +\infty$ limit) in which the effective Hamiltonian dynamics induced by a nonzero tunneling $V_{j,p\alpha} \neq 0$ would take place. We will show that for a suitable choice of the h_{ij} this effective Hamiltonian dynamics is that of higher-spin, possibly many-flavor (depending on the range of α) Kondo model.

We remind the reader that dark states are eigenstates $|D\rangle$ of the Hamiltonian H_{dot}, $H_{\mathrm{dot}} |D\rangle = \varepsilon_D |D\rangle$, which are simultaneously annihilated by all jump operators

L_j, $L_j\,|D\rangle = 0$ [24, 25]. Then, the states $\rho_D = |D\rangle\langle D|$ are stationary states of the dynamics. In our case, following [25], the general construction of the dark states rests only on the strong rotational invariance of the Lindbladian dynamics of the isolated dot:

$$\frac{\mathrm{d}}{\mathrm{d}t}\rho(t) = -\mathrm{i}[H_{\mathrm{dot}}, \rho(t)] + \sum_{j\in\mathrm{dot}} \gamma_j \left(L_j \rho(t) L_j^\dagger - \frac{1}{2}\{L_j^\dagger L_j, \rho(t)\} \right) , \tag{55}$$

in the sense that all components of the total dot spin $S^a = \frac{1}{2}\sum_{j,\sigma\tau}(\sigma^a)_{\sigma\tau} d^\dagger_{j\sigma} d_{j\tau}$ (where σ^a are the Pauli matrices) commute both with the dot Hamiltonian and with all jump operators. Let us diagonalize the Hamiltonian $H_{\mathrm{dot}} = \sum_{a\sigma} \varepsilon_a d^\dagger_{a\sigma} d_{a\sigma}$ in terms of the appropriate single-particle modes $d_{a\sigma} = \sum_j \varphi_a(j) d_{j\sigma}$, where $\varphi_a(j)$ are the eigenfunctions of h_{ij}. Then, we can form an n-particle polarized state $|\uparrow\uparrow\ldots\uparrow\rangle$ in which n fermions with spin $\uparrow$ are put into n different single-body eigenstates of H_{dot} (n must be smaller or equal than the number of dot sites ℓ_d). This state corresponds to a spin eigenstate $|S, M = S\rangle$ where $S = n/2$, and it is trivially a dark state since all fermions have the same spin projection and thus there cannot be any double occupancies. It will have energy $\varepsilon_{\boldsymbol{a}} = \sum_k \varepsilon_{a_k}$, where $\boldsymbol{a} = (a_1, \ldots, a_n)$ is the set of occupied single-body levels. The rest of the states of the spin-S multiplet are generated by repeated application of the ladder operator for the total spin, $S^- \equiv \sum_j d^\dagger_{j\downarrow} d_{j\uparrow} = \sum_a d^\dagger_{a\downarrow} d_{a\uparrow}$ Since $[L_j, S^-] = 0$, the states generated in this way are all annihilated by L_j [3]. These multiplets of many-body eigenstates of a Hamiltonian are known as Dicke states [25–27]. We can see that for any non-extremal choice of the number of particles $0 < n < \ell_d$ (namely, total spin $S = n/2$) there will be $\binom{\ell_d}{n}$ different multiplets with the same spin quantum numbers but different energies (barring degeneracies of $\varepsilon_{\boldsymbol{a}}$ for different $\boldsymbol{a}$), where $\binom{\cdot}{\cdot}$ is the binomial coefficient.

In general, there is no guarantee that the set of dark states just described exhausts the possible stationary states. In the case of a dot consisting of a linear chain of nearest-neighbor hopping fermions (in either periodic or open boundary conditions), with dissipation acting on every site or on just one, we have verified via exact diagonalization (done with the QuTiP package [28, 29]) that this is indeed the case, at least for small chains. For one or two dissipative sites, we have backed up this conclusion with analytical calculations.

We notice that the same construction above can be applied to find the stationary state of the full model, i.e. Eq. (1) in the main text. Namely, one needs to diagonalize the quadratic Hamiltonian (a resonant level model) $H = H_d + H_{\mathrm{leads}} + H_{\mathrm{tun}} = \sum_{a\sigma} \varepsilon_a f^\dagger_{a\sigma} f_{a\sigma}$, and then one can construct the set of Dicke states by filling the single-particle $f_{a\sigma}$ modes. Since, as shown before, the resulting states are free of double occupancies, they are dark states for the two-body losses on the dot site, and the true stationary states for finite-sized leads. However, we expect that the dynamics will take a rather long time to bring the state to the Dicke manifold. In Refs. [30, 31], it was shown that the minimal decay time (i.e. the inverse of the Liouvillian gap) for a fermionic chain of length L with two-body losses on every site scales as L^2. If we restrict the dissipation to act only on one site, the probability of two fermions residing on the dissipative site will be further suppressed, and we can estimate that the longest

[3] Of course, the same set of dark states would be found by starting with the $\downarrow$ polarized state $|\downarrow\downarrow\ldots\downarrow\rangle$ in the same levels $\boldsymbol{a}$, by repeated application of the raising operator $S^+ = (S^-)^\dagger$.

decay time will scale at least as L^3. Since in the NCA that we employed here we take the limit of continuous leads—with infinite particles in them—the Dicke states will never emerge.

The eigenstates of the isolated dot dynamics (55) outside the dark subspace—the bright states—will have a finite dissipation rate, and their population will be depleted in time. For now, we assume that there is a parameter regime in which the decay rate of the all bright states can be made sufficiently large. Later, we will show that for more than one dot site, this requirement does not simply boil down to $\gamma \to +\infty$. In the presence of a large decay rate of the bright states, and in the spirit of adiabatic elimination [3] or the dissipative Schrieffer-Wolff transformation [32], to a first approximation the dynamics will be restricted to the dark subspace, and within this subspace it will be unitary. If we introduce back the coupling to the leads, the latter will mediate transitions between multiplets with neighboring values of S. Indeed, we can write

$$d_{a\sigma} = \sum_{S=0}^{\ell_d/2} \sum_{M=-S}^{S} \sum_{\boldsymbol{b}\in\mathcal{A}_S} \delta^{a\sigma}_{SM\boldsymbol{b}} \, |S-1, M-\sigma, \boldsymbol{b}-(a)\rangle\langle S, M, \boldsymbol{b}| \ , \tag{56}$$

for certain coefficients $\delta^{a\sigma}_{SM\boldsymbol{b}}$. The set $\mathcal{A}_S = \{(a_1, \ldots, a_{2S}) | a_k \neq a_l\}$ is the set of all unordered strings of $2S$ distinct single-particle eigenstates of $H_{\rm dot}$, and the notation $\boldsymbol{b}-(a)$ means that the state a is removed from the string $\boldsymbol{b}$ (if present). Substituting the above expression in $H_{\rm tun}$ we obtain the Hamiltonian in the dark subspace

$$\begin{aligned} H_{\rm eff} = & \sum_{S=0}^{\ell_d/2} \sum_{M=-S}^{S} \sum_{\boldsymbol{a}\in\mathcal{A}_S} \varepsilon_{\boldsymbol{a}} \, |S, M, \boldsymbol{a}\rangle\langle S, M, \boldsymbol{a}| + \sum_{p\sigma\alpha} \varepsilon_{p\alpha} c^\dagger_{p\sigma\alpha} c_{p\sigma\alpha} \\ & + \sum_{p\alpha\sigma a} \sum_{S=0}^{\ell_d/2} \sum_{M=-S}^{S} \sum_{\boldsymbol{a}\in\mathcal{A}_S} (V^{a\sigma}_{SM\boldsymbol{b},p\alpha} \, |S, M, \boldsymbol{b}\rangle\langle S-1, M-\sigma, \boldsymbol{b}-(a)| \, c_{p\sigma\alpha} + \text{H.c.}) \end{aligned} , \tag{57}$$

with $V^{a\sigma}_{SM\boldsymbol{b},p\alpha} \equiv \sum_j V_{j,p\alpha} \varphi^*_a(j) (\delta^{a\sigma}_{SM\boldsymbol{b}})^*$. The above Hamiltonian belongs to the family of the ionic models [2], that have been extensively studied in the context of magnetic impurities in metals. The low-energy description of such models leads to higher-spin Kondo models in suitable regimes. In general, there will be a lowest-energy multiplet with spin S^* (usually, the highest-spin $S^* = \ell_d/2$), separated from the next multiplet by an energy gap $\Delta\varepsilon$. If this gap is much larger than the level width induced by the leads, $\Gamma_\alpha \sim \mathcal{N}_{\alpha F} V^2$ (where $\mathcal{N}_{\alpha F}$ is the single-particle density of states at the chemical potential of lead α, and V is the typical magnitude of the matrix elements $V^{a\sigma}_{SM\boldsymbol{b},p\alpha}$), then $H_{\rm eff}$ can be mapped to a spin S^* Kondo model by the usual Schrieffer-Wolff transformation [1, 2]. On the other hand, there is no constraint on the number of possible leads, thus opening to the possibility of studying over-screened realizations of these exotic Kondo models, that are known to have non-Fermi liquid ground states [2, 33–36].

In any realistic realization of our setup the dissipation rate γ will be finite. Then, the coupling to the leads will introduce a residual dissipation, since by repeated tunneling events into the dot sites one ends up in the dissipated states. The quantitative description of the effective dissipation can be achieved by adiabatic elimination of the dissipated modes [3] or, equivalently, by a dissipative Schrieffer-Wolff transformation [32]. It is simple to guess that the maximal-spin multiplet $S_m = \ell_d/2$ will suffer the largest effective dissipation, since any particle entering the dot will create a double occupancy. Thus, we can expect that the dissipation will couple S_m to $S_m - 1$, with a decay rate $\mathcal{O}\big(V^2/\gamma\big)$, where V is the tunneling rate to the leads. The higher-lying multiplets will need more tunneling events to reach the dissipative states, with a multiplet of spin S acquiring a dissipative rate only at order $S_m - S + 1$ in V^2.

We now consider the concrete scenario of a dot composed of two dissipative sites, for which the dynamics (55) is amenable to an exact solution. We consider the Hamiltonian

$$H_{\mathrm{dot}} = \varepsilon_d \sum_{j=0,\sigma}^{1} d^\dagger_{j\sigma} d_{j\sigma} - J \sum_{\sigma} (d^\dagger_{0\sigma} d_{1\sigma} + \mathrm{H.c.}) = \sum_{a,\sigma} \varepsilon_a d^\dagger_{a\sigma} d_{a\sigma} \ , \tag{58}$$

where the eigenstates are the symmetric and antisymmetric modes $d_{a=\pm,\sigma} = (d_{0\sigma} \pm d_{1\sigma})/2^{1/2}$, corresponding to the energies $\varepsilon_\pm = \varepsilon_d \mp J$. With these states, we obtain the dark subspace multiplet structure shown in Figure 2b in the main text. In detail, we have the $S = 0$ empty dot state $|0\rangle$, the two $S = 1/2$ doublets $d^\dagger_{a\sigma} |0\rangle$ with energies ε_a and the $S = 1$ triplet $\{d^\dagger_{+\uparrow} d^\dagger_{-\uparrow} |0\rangle \,, 2^{-1/2}(d^\dagger_{+\uparrow} d^\dagger_{-\downarrow} + d^\dagger_{+\downarrow} d^\dagger_{-\uparrow}) |0\rangle \,, d^\dagger_{+\downarrow} d^\dagger_{-\downarrow} |0\rangle\}$ with energy $\varepsilon_{S=1} = \sum_a \varepsilon_a = 2\varepsilon_d$. We are considering the case $\varepsilon_d < 0$, $|\varepsilon_d| > J$ which makes the spin triplet the lowest lying multiplet. In this case, following the construction detailed in the previous paragraph, the effective low-energy theory for a weakly coupled bath would yield a $S = 1$ Kondo model. For $-J < \varepsilon_d < 0$, the doublet built out of the symmetric states would have the minimum energy $\varepsilon_d - J$, and the effective low-energy Hamiltonian would be the usual $S = 1/2$ Kondo model.

We now build the full set of bright states of the $\mathcal{L}_{\mathrm{dot}} = -\mathrm{i}[H_{\mathrm{dot}}, \cdot] + \sum_{j\in\mathrm{dot}} \gamma_j \big(L_j \cdot L^\dagger_j - \frac{1}{2}\{L^\dagger_j L_j, \cdot\}\big)$ appearing in Eq. (55). Let us consider the non-Hermitian part of the Lindblad dynamics, $K = H_{\mathrm{dot}} - \mathrm{i}\gamma/2 \sum_j L^\dagger_j L_j = H_{\mathrm{dot}} - \mathrm{i}/2 \sum_j \gamma_j n_{j\uparrow} n_{j\downarrow}$ (with $n_{j\sigma} = d^\dagger_{j\sigma} d_{j\sigma}$ the number operator at site j). For a uniform $\gamma_j = \gamma$, this Hamiltonian is a non-Hermitian version of the Hubbard model [1]. Since K conserves the number of fermions (and spin), we can consider each particle number N_f (and spin S) sector separately. In the one-particle sector, K coincides with H_{dot}, since there are no double occupancies. In the two-particle sectors, it is easy to observe that the eigenstates of $\mathcal{L}_{\mathrm{dot}}$ can be inferred from the diagonalization of K. Indeed, let $K |r_\alpha\rangle = \kappa_\alpha |r_\alpha\rangle$ be the right eigenstates (with $|l_\alpha\rangle$ the left ones, $\langle l_\alpha| K = \kappa_\alpha \langle l_\alpha|$, with the normalization $\langle l_\alpha | r_\beta\rangle = \delta_{\alpha\beta}$). If $L_j |r_\alpha\rangle = 0$ for all j, then $|r_\alpha\rangle$ is one of the dark states built above, and the eigenstates of $\mathcal{L}_{\mathrm{dot}}$ in the dark subspace are $|r_\alpha\rangle\langle r_\beta|$, with eigenvalue $\lambda = -\mathrm{i}(\kappa_\alpha - \kappa_\beta)$ (since dark states are eigenstates of the Hermitian Hamiltonian H_{dot}, right and left eigenstates coincide and the κ_αs are real). This construction of the dark states is valid in all particle-number sectors. Let us consider the $S = 0$, $N_f = 2$ sector with two fermions of opposite spin. Since each jump operator removes precisely two

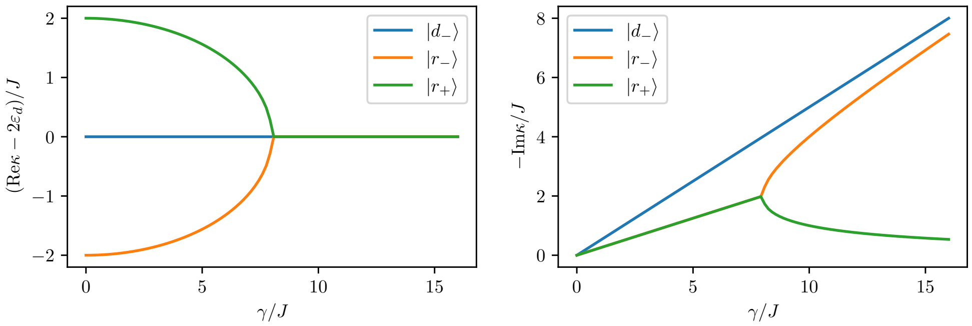

Supplementary Figure 5: Real (left panel) and imaginary parts (right panel) of the eigenvalues of the non-Hermitian Hamiltonian $K = H_{\mathrm{dot}} - \mathrm{i}\gamma/2 \sum_j n_{j\uparrow} n_{j\downarrow}$ in the $N_f = 2$, $S = 0$ sector, showing the presence of a state ($|r_+\rangle$) which becomes effectively dark for infinite dissipation.

opposite-spin fermions, we must have $L_j |r_\alpha\rangle = \eta_{j\alpha} |0\rangle$, and we can anticipate that

$$\sigma = p_0 |0\rangle\langle 0| + p_1 |r_\alpha\rangle\langle r_\beta| \tag{59}$$

will be a right eigenstate of the Lindblad superoperator. Since bright states must be traceless [as $0 = \mathrm{Tr}(\mathcal{L}_{\mathrm{dot}}\sigma) = \lambda \, \mathrm{Tr}\, \sigma$], we have $p_0 = -p_1 \langle r_\beta | r_\alpha\rangle$. Requiring $\mathcal{L}_{\mathrm{dot}}\sigma = \lambda\sigma$ we find

$$\lambda = -\mathrm{i}(\kappa_\alpha - \kappa_\beta^*) \ , \tag{60}$$

provided that $\sum_j \gamma_j \eta_{j\alpha}\eta_{j\beta}^* = -\lambda \langle r_\beta | r_\alpha\rangle$. The latter is indeed satisfied: by taking the overlap of $K|r_\alpha\rangle = \kappa_\alpha |r_\alpha\rangle$ with $|r_\beta\rangle$ we find

$$\langle r_\beta | H_{\mathrm{dot}} | r_\alpha\rangle = \kappa_\alpha \langle r_\beta | r_\alpha\rangle + \frac{\mathrm{i}}{2}\sum_j \gamma_j \eta_{j\alpha}^* \eta_{j\beta} \ . \tag{61}$$

The Hermiticity of H_{dot}, $\langle r_\alpha | H_{\mathrm{dot}} | r_\beta\rangle^* = \langle r_\beta | H_{\mathrm{dot}} | r_\alpha\rangle$ yields the desired relation. From the above discussion, we infer that by diagonalizing K in the two-fermion sector we can find the decay rates $-\mathrm{Re}\,\lambda > 0$ by looking at the imaginary parts of the κ_αs.

We now proceed to diagonalize K in the $N_f = 2$, $S = 0$ sector. We work in real space and we introduce the basis states [4]

$$\begin{cases} |s\rangle = \frac{1}{\sqrt{2}}(|\uparrow,\downarrow\rangle - |\downarrow,\uparrow\rangle) \ , \\ |d_0\rangle = |\uparrow\downarrow, 0\rangle \ , \\ |d_1\rangle = |0, \uparrow\downarrow\rangle \ , \end{cases} \tag{62}$$

where the notation $|\uparrow,\downarrow\rangle = d_{0\uparrow}^\dagger d_{1\downarrow}^\dagger |0\rangle$ denotes a state with the $\uparrow$ fermion sits on the $j = 0$ site while the other sits at the $j = 1$ site, and $|\uparrow\downarrow, 0\rangle$ denotes that both fermions

[4] The "missing" basis state in the subspace with $N_f = 2$ and total magnetization $M = 0$ is the triplet state $|t\rangle = \frac{1}{\sqrt{2}}(|\uparrow,\downarrow\rangle + |\downarrow,\uparrow\rangle)$, which is a dark state belonging to the $S = 1$ manifold.

are at site 0 [5]. In the basis $\{|s\rangle, |d_0\rangle, |d_1\rangle\}$ the non-Hermitian Hamiltonian K reads

$$\begin{pmatrix} 2\varepsilon_d & -\sqrt{2}J & -\sqrt{2}J \\ -\sqrt{2}J & 2\varepsilon_d - \mathrm{i}\gamma_0/2 & 0 \\ -\sqrt{2}J & 0 & 2\varepsilon_d - \mathrm{i}\gamma_1/2 \end{pmatrix} \tag{63}$$

Let us consider the simpler case of a uniform dissipation $\gamma_j = \gamma$. Then, we can form the states $|d_\pm\rangle \equiv (|d_0\rangle \pm |d_1\rangle)/2^{1/2}$ and we find that $K|d_-\rangle = (2\varepsilon_d - \mathrm{i}\gamma/2)|d_-\rangle$. Hence, according to Eq. (60), eigenmatrices of $\mathcal{L}_{\text{dot}}$ involving $|d_-\rangle$ will have a decay rate of at least $\gamma/2$. In the remaining subspace spanned by $\{|s\rangle, |d_+\rangle\}$ we have

$$K = \begin{pmatrix} 2\varepsilon_d & -2J \\ -2J & 2\varepsilon_d - \mathrm{i}\gamma/2 \end{pmatrix}, \tag{64}$$

which is easily diagonalized to find the eigenvalues

$$\kappa_\pm = 2\varepsilon_d - \frac{\mathrm{i}}{4}\gamma \pm \frac{1}{4}(64J^2 - \gamma^2)^{1/2} \tag{65}$$

and the eigenvectors

$$\begin{aligned} |r_\pm\rangle &= [4J^2 + (\kappa_\pm - 2\varepsilon_d)^2]^{-1/2} \begin{pmatrix} 2J \\ -\kappa_\pm + 2\varepsilon_d \end{pmatrix} \\ \langle l_\pm| &= [4J^2 + (\kappa_\pm - 2\varepsilon_d)^2]^{-1/2} \left(2J,\ -\kappa_\pm + 2\varepsilon_d\right) \end{aligned}. \tag{66}$$

Since $K = K^T$ is symmetric, $|l_\pm\rangle = (|r_\pm\rangle)^*$. We sum up the spectral properties of the $N_f = 2$, $S = 0$ sector in Supplementary Figure 5. For the $|r_\pm\rangle$ states we observe two distinct regimes, separated by $\gamma^* = 8J$. This value of γ marks an exceptional point [37] where the two eigenvectors become parallel (and $\kappa_+ = \kappa_-$) and K is no longer diagonalizable. For $\gamma < \gamma^*$, the two eigenvalues $\kappa_\pm$ have distinct real parts while the imaginary part of both is equal to $-\mathrm{i}\gamma/4$. In this regime, a larger dissipation rate corresponds to a larger decay rate for the bright states. For larger $\gamma > \gamma^*$ the two real parts coincide, while the imaginary parts start to diverge from each other: while $-\operatorname{Im}\kappa_- \sim \mathrm{i}\gamma/2 + \mathcal{O}(J^2/\gamma)$ increases further, $-\operatorname{Im}\kappa_-$ *decreases*, ultimately as $8J^2/\gamma$ for large $\gamma \gg J$. This phenomenon can be seen as an incarnation of the Zeno effect, and implies that for $\gamma \to +\infty$ the $|r_+\rangle$ state (which in this limit coincides with the singlet $|s\rangle$) becomes effectively dark. A numerical diagonalization of the Lindbladian with QuTiP confirms that $-2\operatorname{Im}\kappa_+$ is indeed the lowest decay rate at large dissipation. Thus, in order to have an effective dynamics that involves the dark subspace only, we need to increase the intra-dot tunneling J at the same rate as the dissipation γ, i.e. $J \propto \gamma$. At the same time, to have an appreciable Kondo temperature for the effective $S = 1$ (or $S = 1/2$) Kondo model at low energy we must keep the gap $\Delta\varepsilon = |\varepsilon_d + J|$ between the two lowest-lying multiplets finite: this requirement implies that ε_d must be scaled proportionally to γ as well. In other words, the adiabatic elimination of the

[5] We order the labels as $0\uparrow < 0\downarrow < 1\uparrow < 1\downarrow < \dots$.

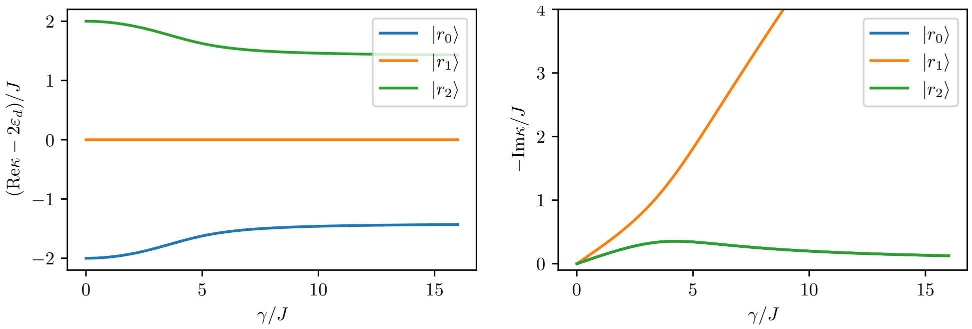

Supplementary Figure 6: Real (left panel) and imaginary parts (right panel) of the eigenvalues of the non-Hermitian Hamiltonian $K = H_{\mathrm{dot}} - \mathrm{i}\gamma/2 n_{0\uparrow} n_{0\downarrow}$ for the case of inhomogeneous dissipation in the $N_f = 2$, $S = 0$ sector, showing the presence of two states ($|r_{1,2}\rangle$) which become effectively dark for infinite dissipation.

bright states is possible only in the regime $|\varepsilon_d| \sim J \sim \gamma \gg \Gamma$, where Γ is the level width induced by the leads. See also the companion paper [23] for further discussions of these points.

To complete our description of the bright states, let us consider the completely filled state $|F\rangle = |\uparrow\downarrow, \uparrow\downarrow\rangle$, which is an eigenstate of K: $K|F\rangle = (4\varepsilon_d - \mathrm{i}\gamma)|F\rangle$. The corresponding eigenmatrix of $\mathcal{L}_{\mathrm{dot}}$ can be constructed by analogy with Eq. (59), namely as a superposition of $|F\rangle\langle F|$, $|r_\alpha^{(2,0,0)}\rangle\langle r_\beta^{(2,0,0)}|$ and $|0\rangle\langle 0|$, where $|r_\alpha^{(N_f,S,M)}\rangle$ indicates a right eigenstate of K in the sector with N_f particles, spin S and magnetization M [6]. We will not reproduce the full calculation here, but it is easy to understand that the real part of the eigenvalue of $\mathcal{L}_{\mathrm{dot}}$ will receive a contribution of $-\mathrm{Im}(\kappa_\alpha + \kappa_\beta)$ from each term $|r_\alpha\rangle\langle r_\beta|$. Hence, the resulting decay rate will be at least 2γ—indeed, the it is the state with the faster decay rate.

Finally, we consider the $N_f = 3$, $S = 1/2$ sector. Since all states in this sector have one unpaired spin, and by particle-hole correspondence with the $N_f = 1$, $S = 1/2$ states, we expect to find two $S = 1/2$ doublets. Indeed, we can think of these states as featuring a "hole" on top of the full state $|F\rangle$: we have verified that the states $|N_f = 3, a\sigma\rangle \equiv d_{-a\sigma}|F\rangle$ are the sought eigenstates, with eigenvalues $\kappa = 4\varepsilon_d - \varepsilon_{-a} + \mathrm{i}\gamma/2$.

We remark that in the present case the diagonalization of K in all symmetry sectors allows to reconstruct all eigenmatrices of the Lindblad superoperator by suitably combining the projectors $|r_\alpha^{(N_f,S,M)}\rangle\langle r_\beta^{(N_f',S',M')}|$, $|r_\alpha^{(N_f-2,S,M)}\rangle\langle r_\beta^{(N_f'-2,S',M')}|$ and $|0\rangle\langle 0|$. As observed above, each term will add a positive contribution $-\mathrm{Im}\,\kappa$ to the decay rate $-\mathrm{Re}\,\lambda$. Since we have proven that the 3- and 4-particle sectors have all $-\mathrm{Im}\,\kappa \propto \gamma$, any time an eigenmatrix features an eigenstate of K with more than 2 particles appears, the decay rate will be linear in γ. The upshot of this discussion is that the lowest decay rate, vanishing for $\gamma \to +\infty$, only comes from the $N_f = 2$, $S = 0$ sector considered before.

[6]Since $|F\rangle$ has $S = M = 0$ and the jump operators conserve spin, the whole eigenmatrix must be composed only of states with the same spin quantum numbers.

The conclusions drawn for the homogeneously dissipated case $\gamma_j = \gamma$ are essentially unchanged also in the completely inhomogeneous one with all dissipation concentrated only on one site, $\gamma_0 = \gamma$, $\gamma_1 = 0$. The dark subspace does not change, while the non-Hermitian Hamiltonian in the $N_f = 2$, $S = 0$ subspace becomes:

$$K = \begin{pmatrix} 2\varepsilon_d & -\sqrt{2}J & -\sqrt{2}J \\ -\sqrt{2}J & 2\varepsilon_d - \mathrm{i}\gamma/2 & 0 \\ -\sqrt{2}J & 0 & 2\varepsilon_d \end{pmatrix}, \tag{67}$$

whose eigenvalues have to be found numerically. The results are shown in Supplementary Figure 6. As in the homogeneous case, there is one mode with $\mathrm{Re}\,\kappa_1 = 2\varepsilon_d$ and a monotonic decay rate $-\mathrm{Im}\,\kappa \sim \gamma/2$, but both the other modes have the same non-monotonic imaginary part—which is generally smaller than in the homogeneous case. The decay rates of these states rises linearly $-\mathrm{Im}\,\kappa \sim \gamma/8$ for small dissipation, reaches a maximum for $\gamma \approx 4J$ and then slowly decreases as $-\mathrm{Im}\,\kappa \sim 2J^2/\gamma$ for large dissipation. In this setup, there is no exceptional point. An exceptional point appears instead in the $N_f = 3$ particle sector, in which

$$K = \begin{pmatrix} 3\varepsilon_d & J \\ J & 3\varepsilon_d - \mathrm{i}\gamma/2 \end{pmatrix} \tag{68}$$

in the basis $\{d_{0\sigma}|F\rangle, d_{1\sigma}|F\rangle\}$ has the same form of Eq. (64) [7]. We see that for a single-site dissipation, also the $N_f = 3$ sector has a state with a non-monotonic decay rate, reaching a maximum for $\gamma = 4J$. The decay rate in this sector is always larger than the one in the $N_f = 2$, $S = 0$ sector, albeit they tend to coincide for large dissipation. The full state $|F\rangle$ has a monotonic decay rate, $K|F\rangle = (4\varepsilon_d - \mathrm{i}\gamma/2)|F\rangle$, which is obviously smaller than in the homogeneous case. This brief analysis of the inhomogeneous setup allows to draw two conclusions: First, a single dissipative site is sufficient to provide all states outside the dark subspace with a finite dissipation rate, and so to implement the mapping to higher-spin Kondo models described above. The second conclusion is that this setup is more fragile than the homogeneous one, in the sense that more bright states become essentially dark for large dissipation $\gamma \gg J$, and that the overall decay rates are smaller than in the homogeneous case.

The ideas presented above can be extended to larger dot sizes, and we expect that the minimal decay rates of the bright states will have a non-monotonic behavior. We have explicitly verified this expectation for $\ell_d = 3$. Thus, in general, the fine-tuning $J \sim |\varepsilon_d| \sim \gamma$ will be needed. This fine-tuning might be circumvented by choosing a different geometry for the dot sites, as for instance a potential well hosting more than one bound state.

References

[1] P. Coleman, *Introduction to Many-Body Physics* (Cambridge University Press, 2015). https://doi.org/10.1017/CBO9781139020916

[7] The matrix reported is identical for the two possible magnetizations $M = \pm 1$.

[2] A.C. Hewson, *The Kondo Problem to Heavy Fermions*. Cambridge Studies in Magnetism (Cambridge University Press, Cambdidge, 1993). https://doi.org/10.1017/CBO9780511470752

[3] J.J. García-Ripoll, S. Dürr, N. Syassen, D.M. Bauer, M. Lettner, G. Rempe, J.I. Cirac, Dissipation-induced hard-core boson gas in an optical lattice. New Journal of Physics **11**(1), 013053 (2009). https://doi.org/10.1088/1367-2630/11/1/013053. URL https://dx.doi.org/10.1088/1367-2630/11/1/013053

[4] N.E. Bickers, Review of techniques in the large-N expansion for dilute magnetic alloys. Rev. Mod. Phys. **59**, 845–939 (1987). https://doi.org/10.1103/RevModPhys.59.845. URL https://link.aps.org/doi/10.1103/RevModPhys.59.845

[5] E.M. Kessler, Generalized schrieffer-wolff formalism for dissipative systems. Phys. Rev. A **86**, 012126 (2012). https://doi.org/10.1103/PhysRevA.86.012126. URL https://link.aps.org/doi/10.1103/PhysRevA.86.012126

[6] S.E. Barnes, New method for the Anderson model. Journal of Physics F: Metal Physics **6**(7), 1375 (1976). https://doi.org/10.1088/0305-4608/6/7/018. URL https://dx.doi.org/10.1088/0305-4608/6/7/018

[7] P. Coleman, New approach to the mixed-valence problem. Phys. Rev. B **29**, 3035–3044 (1984). https://doi.org/10.1103/PhysRevB.29.3035. URL https://link.aps.org/doi/10.1103/PhysRevB.29.3035

[8] L.M. Sieberer, M. Buchhold, S. Diehl, Keldysh field theory for driven open quantum systems. Reports on Progress in Physics **79**(9), 096001 (2016). https://doi.org/10.1088/0034-4885/79/9/096001. URL https://dx.doi.org/10.1088/0034-4885/79/9/096001

[9] M.H. Hettler, J. Kroha, S. Hershfield, Nonequilibrium dynamics of the Anderson impurity model. Phys. Rev. B **58**, 5649–5664 (1998). https://doi.org/10.1103/PhysRevB.58.5649. URL https://link.aps.org/doi/10.1103/PhysRevB.58.5649

[10] N.S. Wingreen, Y. Meir, Anderson model out of equilibrium: Noncrossing-approximation approach to transport through a quantum dot. Phys. Rev. B **49**, 11040–11052 (1994). https://doi.org/10.1103/PhysRevB.49.11040. URL https://link.aps.org/doi/10.1103/PhysRevB.49.11040

[11] N. Sivan, N.S. Wingreen, Single-impurity Anderson model out of equilibrium. Phys. Rev. B **54**, 11622–11629 (1996). https://doi.org/10.1103/PhysRevB.54.11622. URL https://link.aps.org/doi/10.1103/PhysRevB.54.11622

[12] D.C. Langreth, P. Nordlander, Derivation of a master equation for charge-transfer processes in atom-surface collisions. Phys. Rev. B **43**, 2541–2557 (1991). https://doi.org/10.1103/PhysRevB.43.2541. URL https://link.aps.org/doi/10.

1103/PhysRevB.43.2541

[13] H. Shao, D.C. Langreth, P. Nordlander, Many-body theory for charge transfer in atom-surface collisions. Phys. Rev. B **49**, 13929–13947 (1994). https://doi.org/10.1103/PhysRevB.49.13929. URL https://link.aps.org/doi/10.1103/PhysRevB.49.13929

[14] M. Fabrizio, *A Course in Quantum Many-Body Theory*, 1st edn. Graduate Texts in Physics (Springer Cham, 2022)

[15] H. Haug, A.P. Jauho, *Quantum Kinetics in Transport and Optics of Semiconductors*, 2nd edn. Springer Series in Solid-State Sciences (Springer Berlin, Heidelberg, 2007). https://doi.org/https://doi.org/10.1007/978-3-540-73564-9

[16] A. Kamenev, *Field Theory of Non-Equilibrium Systems* (Cambridge University Press, 2011). https://doi.org/10.1017/CBO9781139003667

[17] E. Müller-Hartmann, Self-consistent perturbation theory of the anderson model: ground state properties. Zeitschrift für Physik B Condensed Matter **57**(4), 281–287 (1984)

[18] T.A. Costi, J. Kroha, P. Wölfle, Spectral properties of the anderson impurity model: Comparison of numerical-renormalization-group and noncrossing-approximation results. Phys. Rev. B **53**, 1850–1865 (1996). https://doi.org/10.1103/PhysRevB.53.1850. URL https://link.aps.org/doi/10.1103/PhysRevB.53.1850

[19] Y. Meir, N.S. Wingreen, Landauer formula for the current through an interacting electron region. Phys. Rev. Lett. **68**, 2512–2515 (1992). https://doi.org/10.1103/PhysRevLett.68.2512. URL https://link.aps.org/doi/10.1103/PhysRevLett.68.2512

[20] D. Lobaskin, S. Kehrein, Crossover from nonequilibrium to equilibrium behavior in the time-dependent kondo model. Phys. Rev. B **71**, 193303 (2005). https://doi.org/10.1103/PhysRevB.71.193303. URL https://link.aps.org/doi/10.1103/PhysRevB.71.193303

[21] F.B. Anders, A. Schiller, Real-time dynamics in quantum-impurity systems: A time-dependent numerical renormalization-group approach. Phys. Rev. Lett. **95**, 196801 (2005). https://doi.org/10.1103/PhysRevLett.95.196801. URL https://link.aps.org/doi/10.1103/PhysRevLett.95.196801

[22] M.M. Wauters, C.M. Chung, L. Maffi, M. Burrello, Simulations of the dynamics of quantum impurity problems with matrix product states. Phys. Rev. B **109**, 115101 (2024). https://doi.org/10.1103/PhysRevB.109.115101. URL https://link.aps.org/doi/10.1103/PhysRevB.109.115101

[23] Y.F. Qu, M. Stefanini, T. Shi, T. Esslinger, S. Gopalakrishnan, J. Marino, E. Demler, Variational approach to the dynamics of dissipative quantum impurity models. Phys. Rev. B **111**, 155113 (2025). https://doi.org/10.1103/PhysRevB.111.155113. URL https://link.aps.org/doi/10.1103/PhysRevB.111.155113

[24] H.P. Breuer, F. Petruccione, *The Theory of Open Quantum Systems* (Oxford University Press, 2003)

[25] M. Foss-Feig, A.J. Daley, J.K. Thompson, A.M. Rey, Steady-state many-body entanglement of hot reactive fermions. Phys. Rev. Lett. **109**, 230501 (2012). https://doi.org/10.1103/PhysRevLett.109.230501. URL https://link.aps.org/doi/10.1103/PhysRevLett.109.230501

[26] R.H. Dicke, Coherence in spontaneous radiation processes. Phys. Rev. **93**, 99–110 (1954). https://doi.org/10.1103/PhysRev.93.99. URL https://link.aps.org/doi/10.1103/PhysRev.93.99

[27] F.T. Arecchi, E. Courtens, R. Gilmore, H. Thomas, Atomic coherent states in quantum optics. Phys. Rev. A **6**, 2211–2237 (1972). https://doi.org/10.1103/PhysRevA.6.2211. URL https://link.aps.org/doi/10.1103/PhysRevA.6.2211

[28] J. Johansson, P. Nation, F. Nori, QuTiP: An open-source Python framework for the dynamics of open quantum systems. Computer Physics Communications **183**(8), 1760–1772 (2012). https://doi.org/10.1016/j.cpc.2012.02.021. URL https://doi.org/10.1016/j.cpc.2012.02.021

[29] J. Johansson, P. Nation, F. Nori, QuTiP 2: A Python framework for the dynamics of open quantum systems. Computer Physics Communications **184**(4), 1234–1240 (2013). https://doi.org/10.1016/j.cpc.2012.11.019. URL https://doi.org/10.1016/j.cpc.2012.11.019

[30] M. Nakagawa, N. Kawakami, M. Ueda, Exact Liouvillian Spectrum of a One-Dimensional Dissipative Hubbard Model. Phys. Rev. Lett. **126**, 110404 (2021). https://doi.org/10.1103/PhysRevLett.126.110404. URL https://link.aps.org/doi/10.1103/PhysRevLett.126.110404

[31] H. Yoshida, H. Katsura, Liouvillian gap and single spin-flip dynamics in the dissipative Fermi-Hubbard model. Phys. Rev. A **107**, 033332 (2023). https://doi.org/10.1103/PhysRevA.107.033332. URL https://link.aps.org/doi/10.1103/PhysRevA.107.033332

[32] E.M. Kessler, Generalized Schrieffer-Wolff formalism for dissipative systems. Phys. Rev. A **86**, 012126 (2012). https://doi.org/10.1103/PhysRevA.86.012126. URL https://link.aps.org/doi/10.1103/PhysRevA.86.012126

[33] A.O. Gogolin, A.A. Nersesyan, A.M. Tsvelik, *Bosonization and Strongly Correlated Systems* (Cambridge University Press, Cambridge, 1998)

[34] T. Giamarchi, *Quantum Physics in One Dimension*, 1st edn. (Clarendon Press, Oxford, 2003)

[35] D.B. Karki, C. Mora, J. von Delft, M.N. Kiselev, Two-color Fermi-liquid theory for transport through a multilevel Kondo impurity. Phys. Rev. B **97**, 195403 (2018). https://doi.org/10.1103/PhysRevB.97.195403. URL https://link.aps.org/doi/10.1103/PhysRevB.97.195403

[36] D.B. Karki, A.I. Pavlov, M.N. Kiselev, Multistage Kondo effect in a multiterminal geometry: A modular quantum interferometer. Phys. Rev. B **105**, L041410 (2022). https://doi.org/10.1103/PhysRevB.105.L041410. URL https://link.aps.org/doi/10.1103/PhysRevB.105.L041410

[37] Y. Ashida, Z. Gong, M. Ueda, Non-Hermitian physics. Advances in Physics **69**(3), 249–435 (2020). https://doi.org/10.1080/00018732.2021.1876991. URL https://doi.org/10.1080/00018732.2021.1876991
